# Curcumin promotes spermatogenesis in mice with cryptorchidism by regulating testicular protein *O*-GlcNAcylation

**DOI:** 10.3389/fendo.2025.1555721

**Published:** 2025-06-30

**Authors:** Zhen Wang, Fujia Chen, Yun Li, Chaoying Liu, Lizhen Wang, Weilun Shao, Zhen Lu, Li Hu, Longxuan Li, Yue Wang, Jinyang Lin, Yaxuan Yu, Shengjun Sun, Yurong Yang, Zhijian Zhu, Siqiang Li, Enzhong Li

**Affiliations:** ^1^ School of Biological and Food Processing Engineering, Institute of Agricultural Products Fermentation Engineering and Application, Huanghuai University, Zhumadian, China; ^2^ Biology Institute, Qilu University of Technology (Shandong Academy of Sciences), Jinan, China; ^3^ Aix-Marseille University College, Wuhan University of Technology, Wuhan, China; ^4^ Research & Development (R&D), Henan Dadeguang Animal Pharmaceuticals Co., Ltd., Pingdingshan, Henan, China

**Keywords:** curcumin, spermatogenesis, cryptorchidism, O-GlcNAcylation, testicular protein

## Abstract

**Introduction:**

Curcumin has garnered increasing attention in male reproductive research due to its potential anti-infertility properties. This study aimed to explore the protective effects of curcumin on spermatogenesis impairment in mice with cryptorchidism and to elucidate the underlying mechanisms.

**Methods:**

A total of 56 male Kunming mice aged 10 to 15 days were randomly assigned to different groups, including a control group (BO) and a cryptorchid group with five curcumin treatment groups (CC25, CC50, CC100, CC200, and CC300) receiving varying doses of curcumin (25, 50, 100, 200, and 300 mg/kg, respectively). After five weeks of treatment, evaluations based on organ coefficients, sperm count detection, testicular pathology analysis, and hormone level assessments determined the most effective curcumin dosage.

**Results and disccusion:**

The findings indicated that cryptorchidism had a detrimental impact on reproductive function, evident by decreased testicular coefficient, cessation of sperm production, abnormal testicular tissue morphology, and hormonal imbalances. Curcumin treatment mitigated these abnormalities, with the most significant improvement observed at a dosage of 100 mg/kg, without substantial adverse effects on other organs. Mechanistic studies revealed that cryptorchidism reduced testicular protein *O*-GlcNAcylation levels, while curcumin supplementation effectively increased this modification in a dose-dependent manner. Molecular docking and UDP-GlcNAc analyses further demonstrated that curcumin restores *O-GlcNAc*ylation homeostasis by inhibiting OGA via high-affinity binding and enhancing OGT activity through substrate accumulation, synergistically rebalancing *O-GlcNAc*ylation dynamics. This study uncovers a novel mechanism by which curcumin facilitates spermatogenesis through the regulation of testicular protein *O*-GlcNAcylation, providing a significant theoretical foundation for its utilization in male reproductive health.

## Graphical Abstract

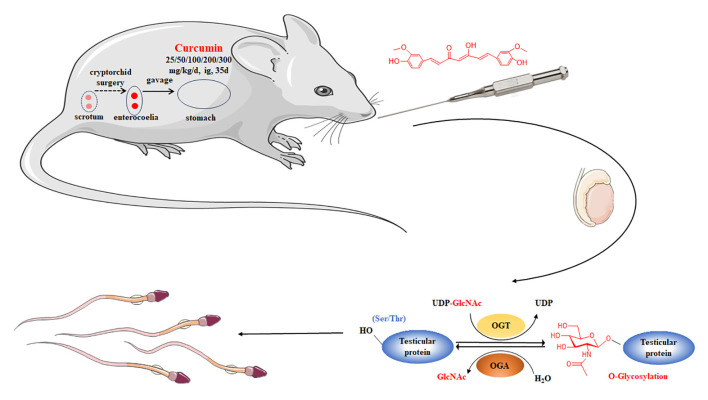

## Highlights

- Curcumin exhibits protective effects against spermatogenesis impairment in mice with cryptorchidism.
- Optimal curcumin dosage for mitigating cryptorchidism-induced abnormalities is determined to be 100 mg/kg.
- Cryptorchidism decreases testicular protein *O*-GlcNAcylation levels, subsequently disrupting spermatogenesis.
- Curcumin enhances testicular protein *O*-GlcNAcylation in a dose-dependent manner, promoting spermatogenesis.
- Study uncovers a novel mechanism of curcumin in promoting male reproductive health via *O*-GlcNAcylation regulation.

## 1 Introduction

Infertility and reproductive disorders have become significant global concerns, with statistics indicating that an estimated 8–12% of couples worldwide experience infertility (1). Recent data from the 2018 “Research Report on the Current Situation of Infertility in China” indicates an increasing incidence of infertility in China, affecting 7%–10% of the population, which amounts to over 40 million individuals (2). The increasing prevalence of infertility, particularly among younger individuals, can be attributed to factors such as increasing environmental pollution and changing lifestyles. Notable, male factors play a significant role in nearly half of all infertility cases, representing 50% of the total cases (1). Therefore, research on male reproductive health is of great importance.

An examination of the factors contributing to male infertility indicates that approximately 40% of cases remain unexplained (3). Identified causes primarily include pre-testicular issues related to hormonal imbalances, testicular complications affecting spermatogenesis (accounting for more than 85% of cases), and post-testicular factors involving abnormalities in sperm transport during epididymal maturation and fertilization (4). Abnormalities in spermatogenesis, the controlled process by which spermatogonial stem cells undergo renewal and differentiation into mature sperm, can manifest as azoospermia, asthenozoospermia, and oligozoospermia, ultimately resulting in male infertility. Herbal medicines and traditional remedies have been widely employed globally to manage various reproductive disorders. These traditional treatments offer distinct advantages over synthetic drugs, including lower toxicity and decreased incidence of adverse reactions (5). Phenolic compounds sourced from medicinal plants offer several health benefits, including anti-inflammatory properties, cancer prevention (6), and the capacity to repair testicular damage and enhance spermatogenesis (7–9).

Curcumin (1,7-bis(4-hydroxy-3-methoxyphenyl)-1,6-heptadiene-3,5-dione), a natural phenolic compound extracted primarily from turmeric, has demonstrated notable anti-inflammatory, antitumor, antiviral, hypoglycemic, and lipid-lowering effects, making it a promising candidate for disease treatment and prevention (10). Recent research has highlighted curcumin’s potential effects on the male reproductive system, including its ability to promote spermatogenesis and enhance reproductive function (11). Nevertheless, the impact of curcumin on the male reproductive system is dosage-dependent. Elevated levels of curcumin can negatively affect sperm concentration, motility, and viability (11). Additionally, high doses of curcumin can decrease testosterone levels and trigger adverse effects in male germ cells, suggesting its potential utility in spermicidal and male contraceptive applications (12, 13). Consequently, determining the optimal dosage of curcumin for different animal models to maximize its beneficial effects on the male reproductive system is a critical research area.

The specific mechanism by which curcumin enhances spermatogenesis is not yet fully understood, although recent international research has yielded significant findings. For example, Tsao et al. found that curcumin can mitigate testicular damage caused by a low-sugar diet, thereby promoting spermatogenesis through its antioxidant properties, enhancement of testosterone production, and inhibition of testicular cell apoptosis and inflammation (14). Zhu and colleagues discovered that curcumin increases the expression of Nrf2 in testicular tissue, leading to the activation of the Nrf2/ARE pathway and the upregulation of downstream antioxidant protein expression, ultimately improving the ability of the testis to combat oxygen free radicals (15). These effects have been demonstrated to decrease testicular ischemia-reperfusion injury and enhance sperm quality (15). Additionally, Wu and co-workers demonstrated that curcumin can alleviate testicular damage caused by hydrogen peroxide in roosters through its antioxidant and anti-apoptotic properties (16). Curcumin enhances spermatogenesis by regulating the expression of Bcl-2, Bax, miRNA-21, and circRNA0001518, leading to a decrease in germ cell apoptosis (17). Moreover, it mitigates tight junction dysfunction in mouse testicular Sertoli cells through the AMPK/SIRT3/mtROS/SOD2 signaling pathway (18). However, additional investigation is necessary to elucidate the exact mechanism underlying curcumin’s promotion of spermatogenesis.

In recent years, there has been a notable increase in research interest surrounding the physiological mechanisms associated with protein glycosylation modification (19–21). This process involves the addition of sugar chains to proteins, catalyzed by a series of glycosyltransferases or glycosidases, leading to the formation of glycoproteins that are essential for numerous physiological functions (19–21). *O*-linked β-N-acetylglucosamine glycosylation (*O*-GlcNAc) is catalyzed by *O*-GlcNAc transferase (OGT), which transfers the GlcNAc moiety from the donor substrate UDP-GlcNAc to the hydroxyl group of Ser/Thr residues, leading to the formation of a β-O-glycosidic bond (22). Conversely, *O*-GlcNAcase (OGA) catalyzes the hydrolysis of this glycosyl modification, enabling the dynamic regulation of *O*-GlcNAc modification (22). Maintaining the homeostasis of *O*-GlcNAc glycosylation is essential for proper physiological functioning, with any disturbance in this equilibrium potentially leading to physiological irregularities and pathological conditions (8). Nevertheless, research is scarce on the *O*-GlcNAc glycosylation of testicular proteins, underscoring the need for additional investigation into its involvement and mechanisms in spermatogenesis.

The current research utilizes a surgical mouse model of cryptorchidism to examine the potential protective properties of different doses of curcumin on spermatogenesis impairment in cryptorchid mice. The study aims to determine the most effective concentration, which will serve as a basis for future clinical investigations. Additionally, the study explores the mechanisms by which curcumin may protect against spermatogenesis abnormalities in cryptorchid mice, focusing on factors such as oxidative stress, hormonal disruptions, changes in testicular protein *O*-GlcNAc glycosylation levels, and associated protein expression.

## 2 Materials and methods

### 2.1 Animals

Fifty-six specific-pathogen-free (SPF) male Kunming mice, aged 10–15 days and weighing 10–15 g, were procured from Huaxing Experimental Animal Farm in Huiji District, Zhengzhou City (License No. SCXK2019-0002). Before the commencement of the experiment, the mice underwent a 3-day acclimation period in a controlled animal room set at a temperature range of 21°C–25°C, with a relative humidity of 50%–65%, and a 12-h light/dark cycle. The mice had access to standard feed and water ad libitum and were housed in a quiet environment. This study was approved by the Animal Ethics Committee of Huanghuai University, and all experimental procedures adhered to the ethical guidelines and regulations set forth by China for the use of animals in research.

### 2.2 Reagents

Anti-*O*-GlcNAc antibodies CTD 110.6 and RL-2 were sourced from Beijing Solarbio Science & Technology Co., Ltd (China) and Cell Signaling (USA), respectively. The Anti-OGT rabbit polyclonal antibody was obtained from Affinity Biosciences (China), MGEA5 was sourced from Invitrogen (USA), and the UAP1 polyclonal antibody was sourced from Proteintech (USA). The Anti-β-actin monoclonal Antibody and related secondary antibodies were purchased from Beijing Solarbio Science & Technology Co., Ltd (China). Additionally, all RT-qPCR-related kits, such as the TaKaRa MiniBEST Universal RNA Extraction Kit (TAKARA 9767), SYBR^®^ Premix Ex Taq™ II (Tli RNaseH Plus, Takara RR820A), and PrimeScript™ RT Master Mix (Perfect Real Time, TaKaRa-RR036Q), were sourced from TaKaRa (China). The ECL chemiluminescent substrate kit, testicular tissue lysate, protein molecular weight marker, and 5× SDS-PAGE loading buffer were procured from Biosharp (China). All other reagents, unless specified otherwise, were obtained from Beijing Solarbio Science & Technology Co., Ltd (China).

### 2.3 Establishment of animal models and grouping

Fifty-six healthy male mice were randomly allocated to seven groups: the normal group (BO), cryptorchidism group (CO), and five curcumin treatment groups, with each group consisting of 8 mice. The mice were individually housed according to their assigned groups. In the BO group, mice underwent sham surgery, were anesthetized with ether, had their abdominal cavities opened and sutured, and were subsequently administered soybean oil via gavage once daily for 35 consecutive days. The CO group underwent a surgical procedure in which mice were anesthetized with ether, positioned supine on an operating table, and their abdominal cavities were incised with a wound measuring approximately 1 cm ± 0.5 mm after being cleaned with 75% alcohol. The testes and epididymis were sutured into the abdominal cavity, indirectly fixing them in place, before the wound was closed and disinfected. Subsequently, soybean oil was administered via gavage once daily for 35 consecutive days starting 24 h post-surgery. The groups receiving curcumin treatment were subjected to the same procedure for inducing cryptorchidism as the control group. Curcumin was administered at varying doses (25, 50, 100, 200, and 300 mg/kg; dissolved in soybean oil) via gavage once daily for 35 days, commencing 24 h after the surgical procedure. These groups were labeled as CC25, CC50, CC100, CC200, and CC300.

### 2.4 Sperm count

After 35 days of treatment, approximately 1 mL of blood samples were collected from the mice’s eyeballs. Subsequently, the mice were euthanized via cervical dislocation, and both testicles were excised and weighed. The left testicle was promptly frozen in liquid nitrogen and stored in an ultra-low temperature freezer at -80°C, while the right testicle was utilized for pathological examination. During the removal of the testicles, the adjacent epididymis was also collected, minced, and placed in a centrifuge tube containing 200 μL of warm PBS. Ten microliters of supernatant from minced epididymis samples were collected and sperm counts were conducted using a Mailang sperm counter. Data analysis was performed using GraphPad Prism 8.04.

### 2.5 Testicular pathology observation

Testicular pathology observations were carried out by fixing the right testes of the mice in Bouin’s fixative for 10 h, followed by dehydration, clearing, embedding, sectioning, and staining with hematoxylin-eosin (H-E). Histopathological changes in the testicular tissue were then observed under a light microscope at 400× magnification.

### 2.6 Analysis of hormone levels in serum

Blood samples were aliquoted into a 1.5 mL EP tube and centrifuged at 2000 g for 20 min after overnight storage at 4°C. Following this, the serum was collected. Enzyme-linked immunosorbent assay kits (Uping Bio, China) were then utilized to measure the concentrations of estradiol (E, product number: YPJ1186), testosterone (T, product number: YPJ1849), follicle-stimulating hormone (FSH, product number: SYP-M0136), luteinizing hormone (LH, product number: SYP-M0400), gonadotropin-releasing hormone (GnRH, product number: SYP-M1938), total superoxide dismutase (T-SOD, product number: SYP-M1740), and malondialdehyde (MDA, product number: YPJ1169) in the serum samples. The procedures outlined in the kit were strictly adhered to during the analysis.

### 2.7 Analysis of testicular protein *O*-GlcNAc glycosylation

Three left testicular tissues from each group of mice were homogenized in testicular tissue lysis buffer and centrifuged at 4°C at 13000 g for 20 min to obtain a testicular tissue homogenate. The total protein concentration of the homogenate was determined using a BCA protein quantitation kit. Subsequently, the proteins were separated by SDS-PAGE and transferred to a nitrocellulose membrane. Primary antibodies RL2 and CTD110.6 were used, with horseradish peroxidase-coupled secondary antibodies utilized for the analysis of *O*-GlcNAcylation. Images were acquired utilizing the ChemiDoc MP system manufactured by Bio-Rad. β-actin and tubulin were employed as internal control proteins. The integrated optical density (IOD) of each band was quantified utilizing Image-Pro Plus 6.0 software developed by Media Cybernetics.

### 2.8 Detection of OGT and OGA expression in testicular tissue

#### 2.8.1 RT-qPCR determination of OGA and OGT mRNA expression

An adequate amount of testicular tissue was extracted from the left testes of three mice in each experimental group. Total RNA was extracted from the tissue samples using the TaKaRa MiniBEST Universal RNA Extraction Kit (TAKARA 9767). The concentration and purity of the RNA were assessed using a NanoDrop 2000 nucleic acid analyzer, ensuring an A260/A280 ratio within the range of 1.8 to 2.0. Subsequently, cDNA was synthesized from mRNA utilizing the SYBR^®^ Premix Ex Taq™ II (Tli RNaseH Plus, Takara RR820A) reverse transcriptase. RT-qPCR was conducted using gene-specific forward and reverse primers in conjunction with PrimeScript™ RT Master Mix (Perfect Real Time, TaKaRa-RR036Q). The primer sequences utilized for the internal reference β-actin were as follows: 5′-CATCCGTAAAGACCTCTATGCCAAC-3′ (upstream) and 5′-ATGGAGCCACCGATCCACA-3′ (downstream). For OGT, the primer sequences were 5′-AAAGATTGCCAGCATGGTTACAG-3′ (upstream) and 5′-GGCCACAGATCTCAGCCTCA-3′ (downstream). Lastly, the primer sequences for OGA were 5′-AACTGTGCCAACAGGACCATC-3′ (upstream) and 5′-GGGCTCCTGGTCTCCAATTAAG-3′ (downstream). The primers were synthesized by Sangon Biotech (Shanghai) Co., Ltd. The PCR reaction conditions included pre-denaturation at 94°C for 30 s, denaturation at 94°C for 5 s, and annealing and extension at 60°C for 30 s, for a total of 40 cycles. The relative expression levels of target gene mRNA were determined using the 2^-ΔΔCt^ method.

#### 2.8.2 Western blot analysis of testicular OGA, OGT, and UAP expression

The experimental procedures mirrored those outlined in section 2.6, with the exception that MGEA5 was employed as the antibody for detecting OGA expression, Anti-OGT rabbit polyclonal antibody was utilized for detecting OGT expression, and UAP1 Polyclonal antibody was employed for detecting UAP1 expression.

### 2.9 Detection of UDP-GlcNAc content

The UDP-GlcNAc content was quantified utilizing a mouse UDP-GlcNAc ELISA kit (product number: RF8887) obtained from Shanghai Ruifan Biotechnology Co., Ltd. The experimental procedures were conducted per the manufacturer’s instructions. Specifically, samples (mouse serum or testicular tissue homogenate), UDP-GlcNAc standards, and HRP-labeled detection antibodies were successively applied to the pre-coated microwells containing UDP-GlcNAc antibodies. Following incubation and extensive washing steps, the substrate tetramethylbenzidine (TMB) was employed for chromogenic detection. The optical density (OD) was determined at 450 nm using an enzyme-linked immunosorbent assay. The concentration of UDP-GlcNAc in the mouse testicular homogenate was measured using a kit method (in ng/mL), and subsequently, the concentration of UDP-GlcNAc per unit mass of mouse testis (in ng/g) was calculated based on the mass coefficient of the testicular homogenate (in g/mL, which is the ratio of the mass of the mouse testis to the volume of the lysis solution).

### 2.10 Molecular docking analysis

To analyze the binding affinities and modes of interaction between the drug(curcumin) and their targets(OGA and OGT), AutodockVina 1.2.2, a silico protein–ligand docking software was employed (23). The molecular structures of curcumin (PubChem CID: 969516) was retrieved from PubChem Compound (https://pubchem.ncbi.nlm.nih.gov/) (24). The 3D coordinates of OGA (PDB ID, 5UHP; resolution, 2.79 Å) and OGT(PDB ID, 6EOU; resolution, 1.75 Å) were downloaded from the PDB (http://www.rcsb.org/pdb/home/home.do). For docking analysis, all protein and molecular files were converted into PDBQT format with all water molecules excluded and polar hydrogen atoms were added. The grid box was centered to cover the domain of each protein and to accommodate free molecular movement. The grid box was set to 30 Å × 30 Å × 30 Å, and grid point distance was 0.05nm. Molecular docking studies were performed by Autodock Vina 1.2.2 (http://autodock.scripps.edu/).

### 2.11 Statistical analysis

Statistical analyses were performed using GraphPad Prism 8.0.2 (Boston, MA, USA). Intergroup comparisons were analyzed by Student’s t-test, with a P-value < 0.05 considered statistically significant and a P-value < 0.01 indicating highly statistically significant differences.

## 3 Results

### 3.1 Confirmation of stall and recovery of spermatogenesis in mice

#### 3.1.1 Effects of curcumin on body weight and organ coefficients in cryptorchid mice

The study demonstrated that cryptorchid surgery resulted in decreased appetite, reduced activity, and dull hair in mice, which subsequently returned to baseline levels within 24 h. Throughout the experiment, all mice exhibited smooth fur, high levels of activity, and good mental health, with no observable abnormalities. Analysis of the data presented in **Table 1** indicated that there was no statistically significant variance in the initial weight of mice across the experimental groups before the commencement of the study (P > 0.05). The body weight of mice in each group exhibited a consistent increase post-surgery, with no statistically significant differences observed during the initial three weeks. However, from the fourth week onwards, there was still no significant change in body weight between the BO and CO groups, suggesting that cryptorchid surgery did not have a significant impact on the body weight of mice (**Table 1**). Additionally, compared to the CO group, mice in the CC50, CC100, and CC200 groups exhibited significantly higher body weights (P < 0.01), whereas the CC25 and CC300 groups did not show a significant increase. Notably, the body weights of mice in the CC50, CC100, and CC200 groups significantly increased compared to the control group (CO) (P < 0.01), while the CC25 and CC300 groups did not show a statistically significant change (P > 0.05). These findings suggest that the administration of curcumin may have an impact on the growth and development of mice in a dose-dependent manner.

**Table 1:** Effects of cryptorchid surgery and curcumin administration on body weight in Mice (n=8, mean ± standard deviation, g).

Group	0 d	7 d	14 d	21 d	28 d	35 d
BO	17.87 ± 1.50	25.52 ± 1.86	29.55 ± 1.49	32.08 ± 1.92	34.01 ± 1.70	35.39 ± 1.74
CO	17.12 ± 2.15	23.58 ± 2.47	29.47 ± 2.60	34.67 ± 2.30	34.47 ± 2.56	35.87 ± 3.00
CC25	17.70 ± 1.06	25.61 ± 1.12	3ifg0.50 ± 1.40	34.19 ± 2.44	35.40 ± 2.29	35.15 ± 4.12
CC50	17.92 ± 1.54	26.33 ± 1.17	29.99 ± 2.54	34.63 ± 1.67	36.79 ± 1.75*	40.84 ± 2.35**
CC100	17.97 ± 0.66	27.25 ± 1.12	32.18 ± 0.94	35.08 ± 1.20	37.12 ± 1.30*	40.67 ± 1.73**
CC200	17.89 ± 1.28	27.70 ± 1.42	32.30 ± 1.61	35.19 ± 1.72	36.18 ± 1.56*	40.38 ± 1.73**
CC300	17.96 ± 1.56	26.54 ± 1.95	30.86 ± 2.45	33.34 ± 2.41	34.85 ± 1.49	34.06 ± 1.02

*Compared to the CO control group, P < 0.05; **Compared to the CO control group, P < 0.01.

Following the experiment, the organ coefficients (organ weight/body weight, mg/g) of various organs in mice were assessed. There were no statistically significant alterations in the coefficients of the brain, cerebellum, heart, kidney, spleen, stomach, seminal vesicle, lungs, and colon, suggesting that cryptorchid surgery and curcumin administration did not have a significant impact on the growth of these organs (**Supplementary Figure S1**). However, analysis of the testicular coefficient indicated a significant decrease in the CO group compared to the BO group (P < 0.01), implying that cryptorchid surgery, which involves the relocation of the testes to the abdominal cavity, has a notable effect on testicular development (**Supplementary Figure S1A**).

Compared to the control group, mice treated with curcumin exhibited a non-significant increase in testicular coefficient in the CC25, CC50, CC100, and CC200 groups (P > 0.05), while a significant increase was observed in the CC300 group (P < 0.01). These findings suggest that curcumin may impact testicular growth and development in mice, with a notable promotion of testicular development at a high dose of curcumin (300 mg/kg) (**Supplementary Figure S1A**). Analysis of the liver coefficient revealed no significant difference between the control and cryptorchid groups (P > 0.05), indicating that cryptorchid surgery does not have a significant effect on the liver coefficient (**Supplementary Figure S1G**). Compared to the control group (CO), mice administered with curcumin displayed changes in liver coefficient; however, these alterations were not statistically significant (P > 0.05). Notably, a decrease in liver coefficient was observed in the CC200 (P < 0.05) and CC300 (P < 0.01) groups compared to the CC25, CC50, and CC100 groups, suggesting a potential impact of curcumin on liver growth and development in mice (**Supplementary Figure S1G**). Analysis of the pancreas coefficient indicated no statistically significant difference between the cryptorchid (CO) group and the bilateral orchiectomy (BO) group (P > 0.05), suggesting that cryptorchid surgery does not have a significant impact on the development of the pancreas (**Supplementary Figure S1J**). Furthermore, a decrease in pancreas coefficient was observed in all mice treated with curcumin compared to the CO group, with statistically significant changes only evident in the CC50 and CC300 groups (P < 0.05). This finding suggests that curcumin may influence pancreatic growth and development in mice in a dose-dependent manner (**Supplementary Figure S1J**).

#### 3.1.2 Effect of curcumin on sperm count in cryptorchid mice

**Figure 1:**
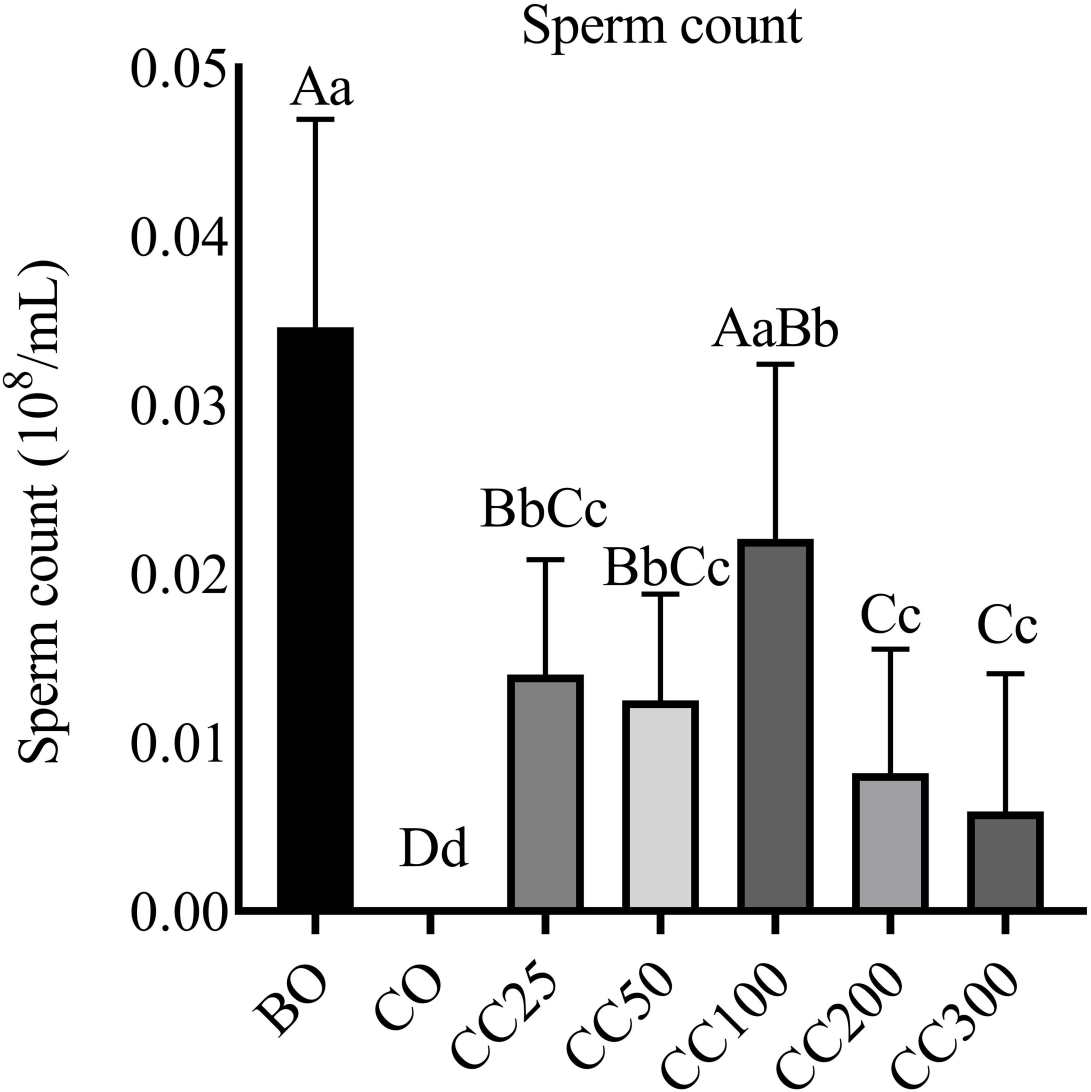
Effects of curcumin on sperm count in mice. Columns with different uppercase and lowercase letters indicate extremely significant differences (P < 0.01), columns with the same uppercase letters but different lowercase letters indicate significant differences (P < 0.05), and those with identical letters indicate no significant difference (P > 0.05).

Following the experimental procedure, the epididymis of the mice was extracted to measure sperm count. The findings revealed a lack of sperm in the CO group, confirming the successful establishment of the cryptorchidism model (**Figure 1**). Compared to the CO group, supplementation with varying concentrations of curcumin notably elevated sperm density (P < 0.01), indicating the potential of curcumin to enhance spermatogenesis in cryptorchid mice and mitigate abnormal spermatogenesis resulting from cryptorchidism. The impact of different doses of curcumin supplementation on sperm density varied. Specifically, no statistically significant difference in sperm count was observed when curcumin was administered at concentrations ranging from 25 to 200 mg/kg (P > 0.05). However, at a concentration of 300 mg/kg, a significant decrease in sperm count was noted compared to the 100 mg/kg group (P < 0.01), although no significant variance was detected when compared to other curcumin concentrations (P > 0.05). Furthermore, the sperm count of the CC100 group was lower than that of the BO group, although this difference was not significant (P > 0.05). Conversely, the sperm counts of the remaining curcumin-supplemented groups exhibited a statistically significant decrease compared to the BO group (P < 0.01). These findings suggest that curcumin supplementation at a dose of 100 mg/kg yields the most favorable outcome in enhancing sperm density in mice.

#### 3.1.3 Effects of curcumin on testicular histology in cryptorchid mice

**Figure 2:**
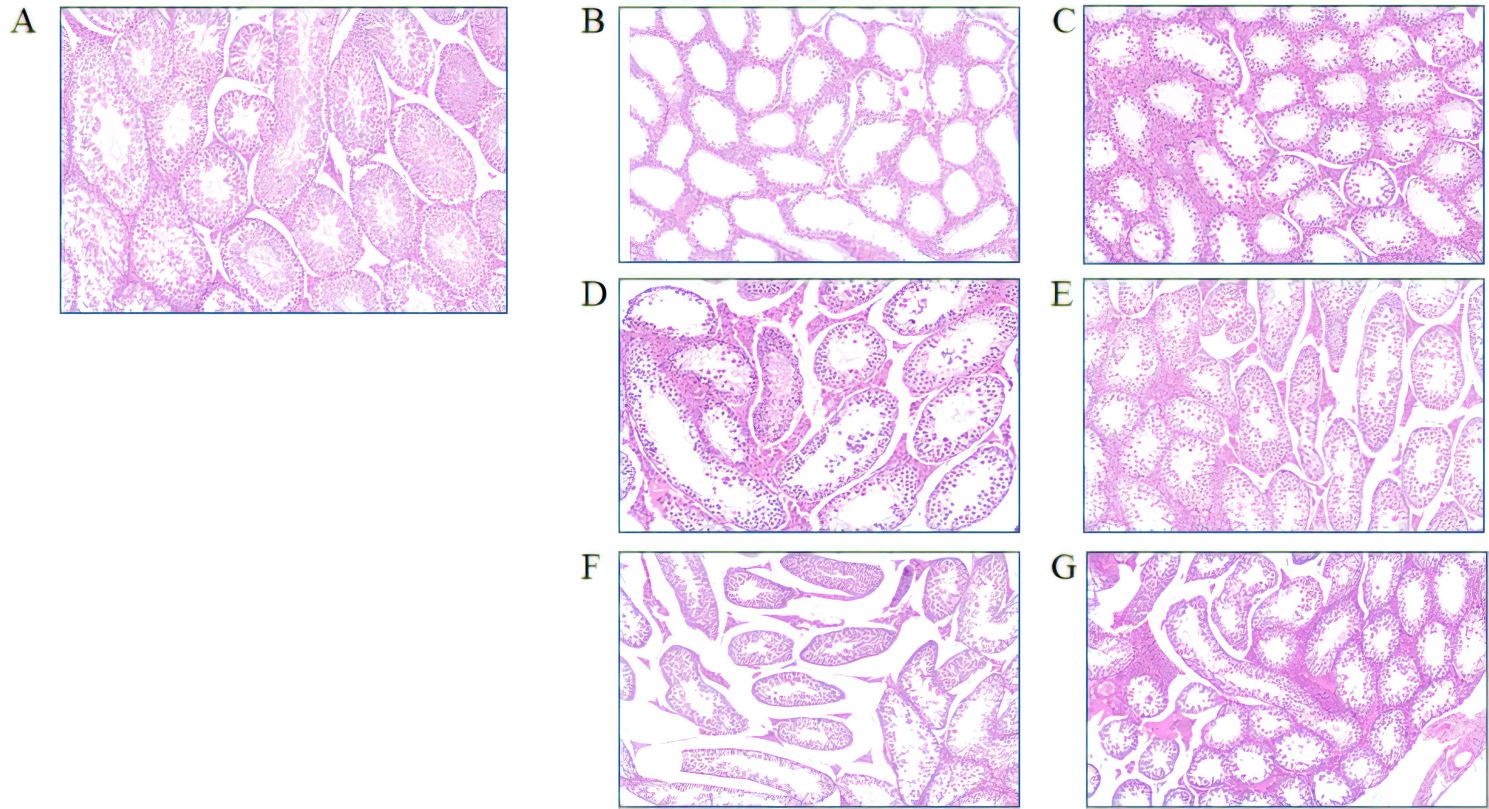
Effects of curcumin on testicular histology caused by cryptorchid surgery, BO Group **(A)**, CO Group **(B)**, CC25 Group **(C)**, CC50 Group **(D)**, CC100 Group **(E)**, CC200 Group **(F)**, CC300 Group **(G)**.

After the experiment, the right testes of the mice were collected for pathological examination. In the BO group, the seminiferous tubules of the mouse testes exhibited a well-developed structure, characterized by a dense arrangement of spermatogenic cells within the spermatogenic epithelium and a substantial presence of spermatozoa within the lumen (**Figure 2A**). Following cryptorchid surgery, notable morphological alterations were observed in the mouse testes, including seminiferous tubule atrophy, disruption of the spermatogenic epithelium, azoospermia, diminished spermatogonia population, and germ cell shedding (**Figure 2B**). However, these adverse histological changes resulting from cryptorchid surgery were significantly mitigated following the administration of curcumin. In the CC25, CC50, and CC100 groups, notable improvements were observed in the morphology of the seminiferous tubules in mouse testes, including thicker walls, well-arranged germ cells at various stages of development, smaller lumens, larger tube diameters, and the presence of mature sperm cells (**Figures 2C–E**). Specifically, compared to the control group (BO), the lumens of the seminiferous tubules in the CC25 and CC50 groups were larger, with a reduced number of mature sperm cells present. However, no significant difference was observed between the CC100 and control groups.

The presence of mature sperm in the seminiferous tubules of the CC200 and CC300 groups suggests a protective effect against cryptorchidism-induced testicular damage. However, the recovery from such damage was limited, with persistent structural damage observed in the seminiferous tubules (**Figures 2F–G**). These findings indicate that varying doses of curcumin offer a degree of protection against testicular damage following cryptorchid surgery. Specifically, within the range of 25 to 100 mg/kg of curcumin, higher concentrations of the compound demonstrated enhanced protective effects. At a dosage of 100 mg/kg, curcumin demonstrated significant efficacy in repairing damage resulting from cryptorchid surgery. However, with further dosage increases, the protective effects of curcumin on testicular health diminished.

### 3.2 Effects of curcumin on serum hormone levels in mice with cryptorchidism

**Figure 3:**
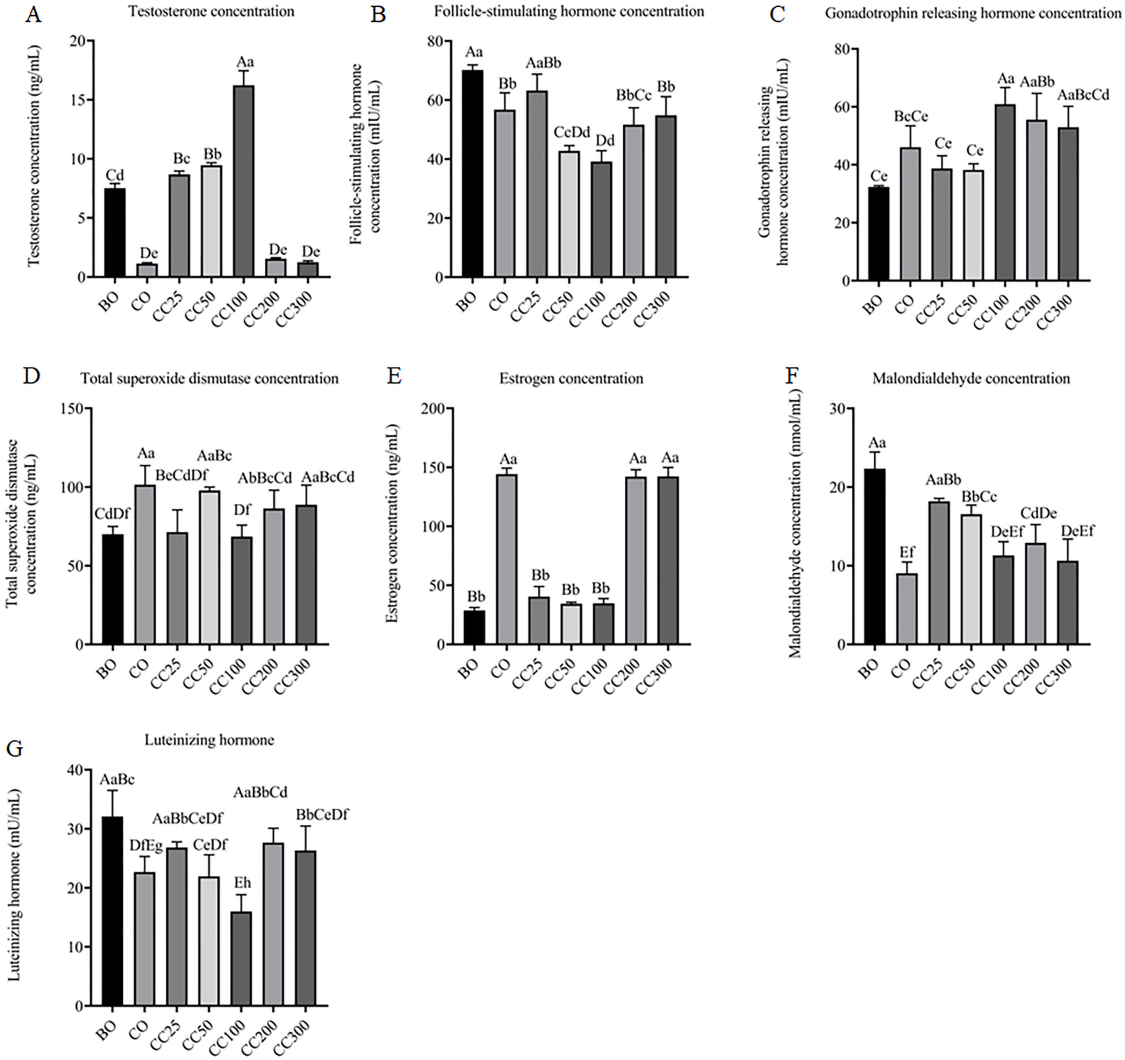
Effects of curcumin on serum hormone level defects caused by cryptorchid surgery: testosterone **(A)**, follicle-stimulating hormone **(B)**, gonadotropin-releasing hormone **(C)**, total superoxide dismutase **(D)**, estrogen **(E)**, malondialdehyde **(F)**, luteinizing hormone **(G)**. Columns with different uppercase and lowercase letters indicate extremely significant differences (P < 0.01), columns with the same uppercase letters but different lowercase letters indicate significant differences (P < 0.05), and completely identical letters indicate non-significant differences (P > 0.05).

The male reproductive function is regulated by a complex interplay of hormones within the hypothalamic-pituitary-testicular axis, and alterations in hormone levels can impact male fertility (25). ELISA was utilized in this research to assess the hormone levels in the serum of mice across various groups. The hormones assessed included testosterone, follicle-stimulating hormone, gonadotropin-releasing hormone, total superoxide dismutase, estrogen, malondialdehyde, and luteinizing hormone. The findings indicated that both cryptorchid surgery and curcumin intervention at varying dosages exerted specific impacts on hormone levels (**Figure 3**).

Testosterone levels were positively correlated with spermatogenesis in male mice (26, 27). As shown in **Figure 3A**, serum testosterone levels in the CO group were significantly reduced compared to the BO group (P < 0.01), indicating that cryptorchidism leads to a decrease in internal testosterone levels. In contrast, testosterone levels in the CC25, CC50, and CC100 groups were significantly increased compared to the CO group (P < 0.01), and their concentrations exceeded those in the BO group (P < 0.01), indicating that curcumin intervention at doses of 50–100 mg/kg can increase testosterone levels in mice with cryptorchidism in a dose-dependent manner. However, there was no significant change in testosterone levels in the CC200 and CC300 groups compared to the CO group, indicating that high-dose curcumin (≥200 mg/kg) cannot significantly increase testosterone levels.

Low concentrations of follicle-stimulating hormone (FSH) can promote the generation of seminiferous tubules, but excessively high concentrations can inhibit testosterone levels, which is detrimental to spermatogenesis (28). As shown in **Figure 3B**, the level of FSH in the serum of male mice in the CO group was significantly reduced compared to the BO group (P < 0.01), indicating that cryptorchidism leads to a decrease in internal FSH levels. We speculate that the serum FSH concentration in cryptorchid mice (CO group) was significantly lower than that in sham-operated mice (BO group), potentially indicating a compensatory response to cryptorchidism-induced damage. This reduction in FSH may reflect an attempt by the body to stimulate seminiferous tubule formation. Interestingly, curcumin treatment at doses of 50–100 mg/kg further reduced FSH levels compared to the CO group (P < 0.01), suggesting that curcumin may modulate the FSH to promote testicular repair. However, this effect was not observed in mice treated with lower doses of curcumin, indicating a dose-dependent effect. These findings suggest that curcumin may exert its protective effect on testicular function by influencing the hypothalamic-pituitary-gonadal axis, potentially enhancing the repair of seminiferous tubules and spermatogenesis.

The gonadotropin-releasing hormone can promote the secretion of testosterone, which is beneficial for spermatogenesis (27). Compared to the BO group, the serum gonadotropin-releasing hormone content in the CO group increased, but change was not statistically significant (P > 0.05), indicating that cryptorchid surgery did not lead to significant changes in the level of gonadotropin-releasing hormone in the body (**Figure 3C**). However, compared to the CO group, the gonadotropin-releasing hormone levels in the CC100 and CC200 groups were significantly increased (P < 0.01), while no significant changes were observed in the other groups. These findings suggest that curcumin intervention at doses of 100–200 mg/kg may increase testosterone levels by elevating gonadotropin-releasing hormone levels in mice with cryptorchidism, thereby repairing the damage caused by cryptorchid surgery.

Total superoxide dismutase level represents the antioxidant capacity of mouse cells and negatively correlates with oxidative stress (29). Compared to the BO group, the serum level of total superoxide dismutase in the CO group increased (P < 0.01), indicating severe oxidative stress in mice in the CO group (**Figure 3D**). Compared to the CO group, the total superoxide dismutase levels in the CC25, CC100, and CC200 groups were significantly reduced (P < 0.05), while no significant change was observed in the other groups. These findings indicate that curcumin, as an exogenous antioxidant drug, can reduce endogenous total superoxide dismutase levels in mice and improve the oxidative state in mice (**Figure 3D**).

High levels of estradiol negatively affect sperm quantity and quality, reducing male fertility (30). Compared to the BO group, the serum estradiol level in mice in the CO group was significantly increased (P < 0.01), indicating severe impairment of spermatogenesis in mice with cryptorchidism (**Figure 3E**). Compared to the CO group, the estradiol levels in the CC25, CC50, and CC100 groups were significantly reduced (P < 0.01), while no significant changes were observed in other groups (**Figure 3E**). These findings suggest that curcumin intervention at doses of 25–100 mg/kg may improve spermatogenesis in mice by reducing estradiol levels. However, high-dose curcumin (≥200 mg/kg) did not reduce estradiol levels in mice.

Malondialdehyde (MDA) is a marker of lipid peroxidation, which occurs during oxidative stress and can be detrimental to sperm cells (31). Surprisingly, compared to the sham-operated group (BO), the CO group exhibited significantly lower serum MDA levels (**Figure 3F**), suggesting an effective antioxidant response to cryptorchidism-induced damage. This was further supported by the increased total superoxide dismutase (SOD) levels in the CO group, indicating enhanced antioxidant capacity (**Figure 3D**). Compared to the CO group, the malondialdehyde levels significantly increased in the CC25, CC50, and CC200 groups (P < 0.01), while no significant changes were observed in the other experimental groups. These findings indicate that curcumin can increase MDA levels in mice and improve spermatogenesis in a dose-dependent manner.

The luteinizing hormone promotes the secretion of testosterone and works synergistically with follicle-stimulating hormone to maintain normal spermatogenesis (32). Compared to the BO group, the serum luteinizing hormone level in mice in the CO group was significantly reduced (P < 0.01), indicating that cryptorchid surgery can significantly reduce luteinizing hormone levels (**Figure 3G**), consistent with changes in testosterone levels (**Figure 3A**). Compared to the CO group, the luteinizing hormone level of the CC100 group was significantly reduced (P < 0.05), while the luteinizing hormone level in mice in the CC200 group significantly increased (P < 0.01). No significant changes in luteinizing hormone levels were observed in the other experimental groups (**Figure 3G**).

In summary, cryptorchid surgery can disrupt the reproductive internal environment of mice, interfere with the secretion of multiple reproductive hormones in the hypothalamic-pituitary-testicular axis of males, impair spermatogenesis, and alter the levels of hormones related to mouse reproduction. Curcumin can dose-dependently improve these hormone level disruptions caused by cryptorchid surgery, promoting spermatogenesis in mice. Notably, 100 mg/kg may be the optimal curcumin dose to promote spermatogenesis.

### 3.3 Effects of curcumin on testicular protein *O*-GlcNAcylation in mice with cryptorchidism

**Figure 4:**
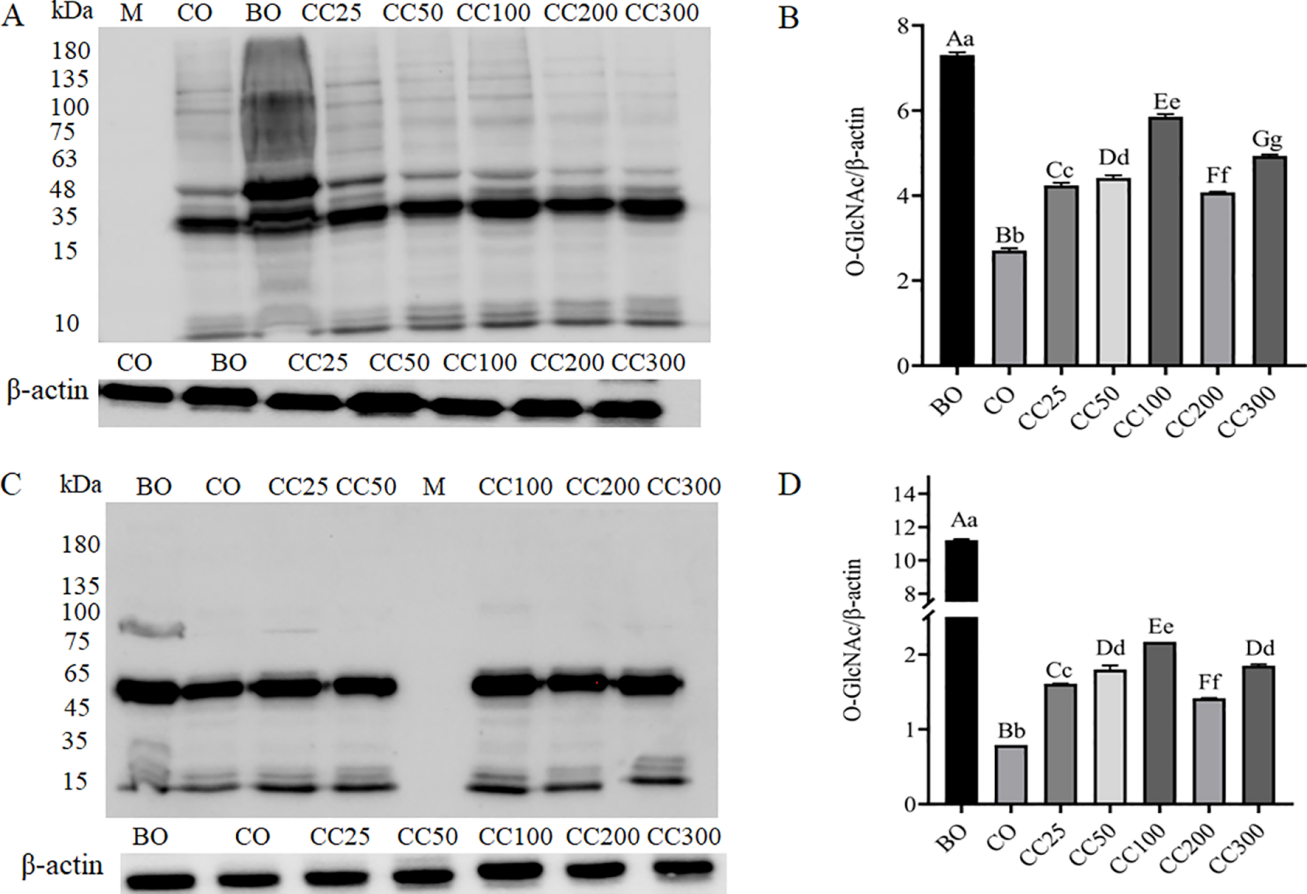
Effects of curcumin on testicular protein *O*-GlcNAcylation caused by cryptorchid surgery, detected using commercial *O*-GlcNAc antibodies RL.2 **(A)** and CTD110.6 **(C)**. **(B, D)** represent the relative average density analysis of Western Blot, respectively. Columns with different uppercase and lowercase letters indicate extremely significant differences (P < 0.01), columns with the same uppercase letters but different lowercase letters indicate significant differences (P < 0.05), and completely identical letters indicate non-significant differences (P > 0.05).

To investigate the effects of curcumin on testicular protein *O*-GlcNAcylation induced by cryptorchid surgery, total protein was extracted from a mixture of three left testicular tissues selected from each group of mice in this study. Western blot analysis was performed on the total testicular protein using commercial antibodies RL2 (**Figures 4A, B**) and CTD110.6 (**Figures 4C, D**).

The results presented in **Figures 4A, B** indicate that, compared to the BO group, testicular protein *O*-GlcNAcylation was significantly reduced in the CO group, consistent with our previous report (8). After supplementation with different doses of curcumin, the *O*-GlcNAcylation of testicular protein was significantly higher than that in the CO group. Notably, the CC100 group had the highest expression, followed by the CC300, CC50, CC25, and CC200 groups. **Figures 4C, D** further demonstrate that, compared to the BO group, testicular protein *O*-GlcNAcylation was significantly reduced in the CO group, underscoring the relationship between spermatogenesis disorders in cryptorchid mice and the marked reduction in testicular protein *O*-GlcNAcylation. Meanwhile, **Figures 4C, D** further validate the effects of different doses of curcumin supplementation on testicular protein *O*-GlcNAcylation. These results suggest that the significant reduction in testicular protein *O*-GlcNAcylation in cryptorchid mice contributes to spermatogenesis disorders. Curcumin improves spermatogenesis damage caused by cryptorchid surgery by dose-dependently upregulating the level of testicular protein *O*-GlcNAcylation. In addition to total protein *O*-GlcNAcylation, the *O*-GlcNAcylation of certain protein bands in the cryptorchidism group, such as bands of approximately 35 kDa, 48 kDa, 100 kDa, and 63 kDa, was reduced compared to the control group. The *O*-GlcNAcylation of these proteins further changed in the curcumin-treated cryptorchidism group in a dose-dependent manner, indicating that the *O*-GlcNAcylation of these proteins may be closely related to spermatogenesis.

### 3.4 Effects of curcumin on UDP-GlcNAc concentration in the serum and testes of mice with cryptorchidism

**Figure 5:**
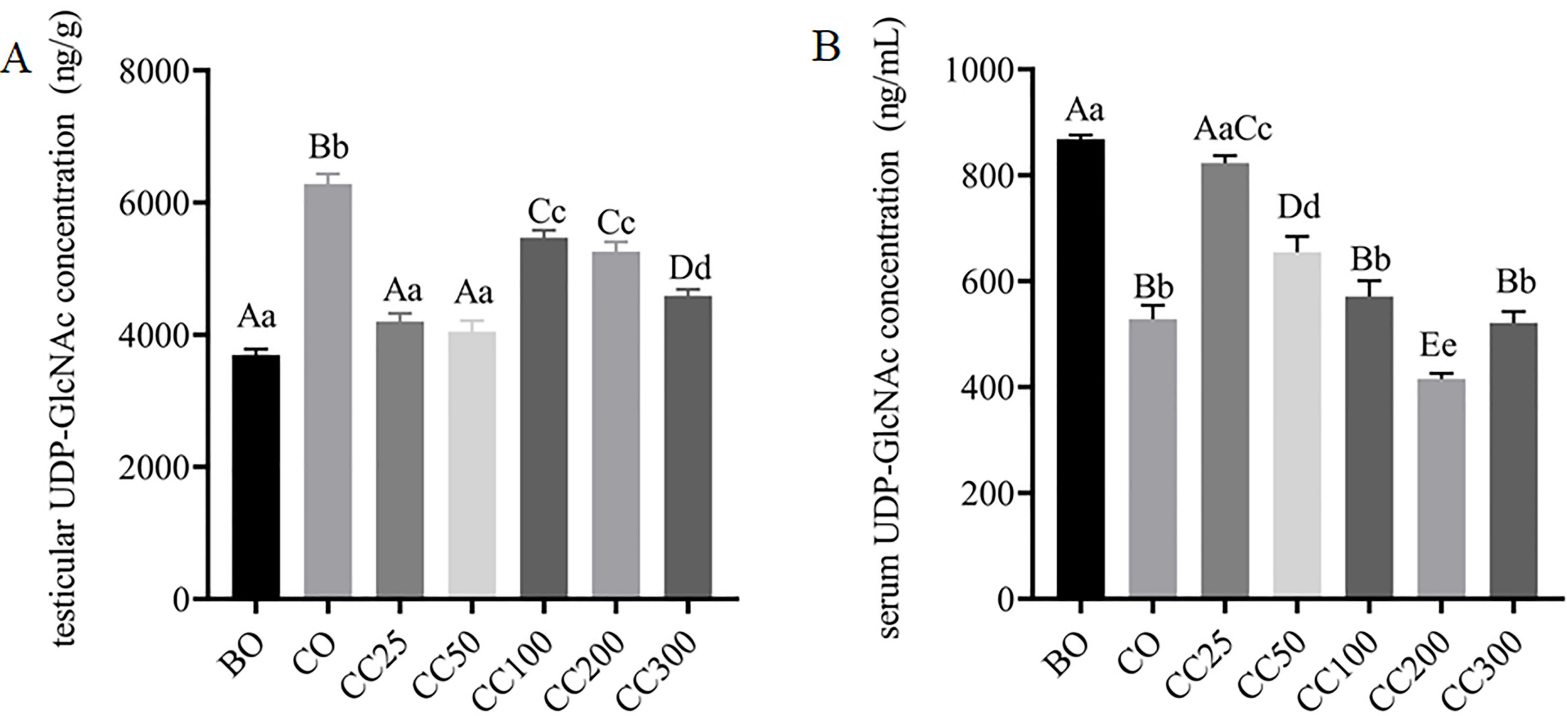
Effects of curcumin on UDP-GlcNAc concentration in testes **(A)** and serum **(B)** following cryptorchid surgery. Columns with different uppercase and lowercase letters indicate extremely significant differences (P < 0.01), columns with the same uppercase letters but different lowercase letters indicate significant differences (P < 0.05), and completely identical letters indicate non-significant differences (P > 0.05).

UDP-N-acetylglucosamine (UDP-GlcNAc), a donor substrate, directly affects the level of *O*-GlcNAcylation. The UDP-GlcNAc concentration in testicular tissues from mice in the CO group was significantly reduced compared to those from mice in the BO group (P < 0.001), indicating that cryptorchid surgery significantly reduces testicular UDP-GlcNAc concentration. Supplementation with curcumin increased the testicular UDP-GlcNAc concentration in the mice. Notably, the testicular UDP-GlcNAc concentration in the CC25, CC50, CC100, and CC200 groups increased significantly (**Figure 5A**), though it remained lower than that in the BO group. The increase in testicular UDP-GlcNAc concentration in the CC300 group was not significant, indicating that curcumin supplementation can increase testicular UDP-GlcNAc concentration in mice, with the extent of increase inversely related to the curcumin dose. Additionally, the serum UDP-GlcNAc concentration in the CO group was significantly reduced compared to the BO group (P < 0.001), indicating that cryptorchid surgery significantly reduces serum UDP-GlcNAc concentration. The serum UDP-GlcNAc concentration in the CC25 and CC50 groups increased significantly (**Figure 5B**). Meanwhile, there was no significant difference in serum UDP-GlcNAc concentration between the CC25 and BO groups. However, the serum UDP-GlcNAc concentration in the CC200 group was significantly lower than that in the CO group, while no significant changes in serum UDP-GlcNAc concentrations were observed in the CC100 and CC300 groups (**Figure 5B**). These findings suggest that an appropriate amount of curcumin can increase serum UDP-GlcNAc concentration in mice, while excessive curcumin concentration may decrease serum UDP-GlcNAc concentration.

### 3.5 Effects of curcumin on the expression of proteins related to *O*-GlcNAc glycosylation modification in the testes of cryptorchid mice

**Figure 6:**
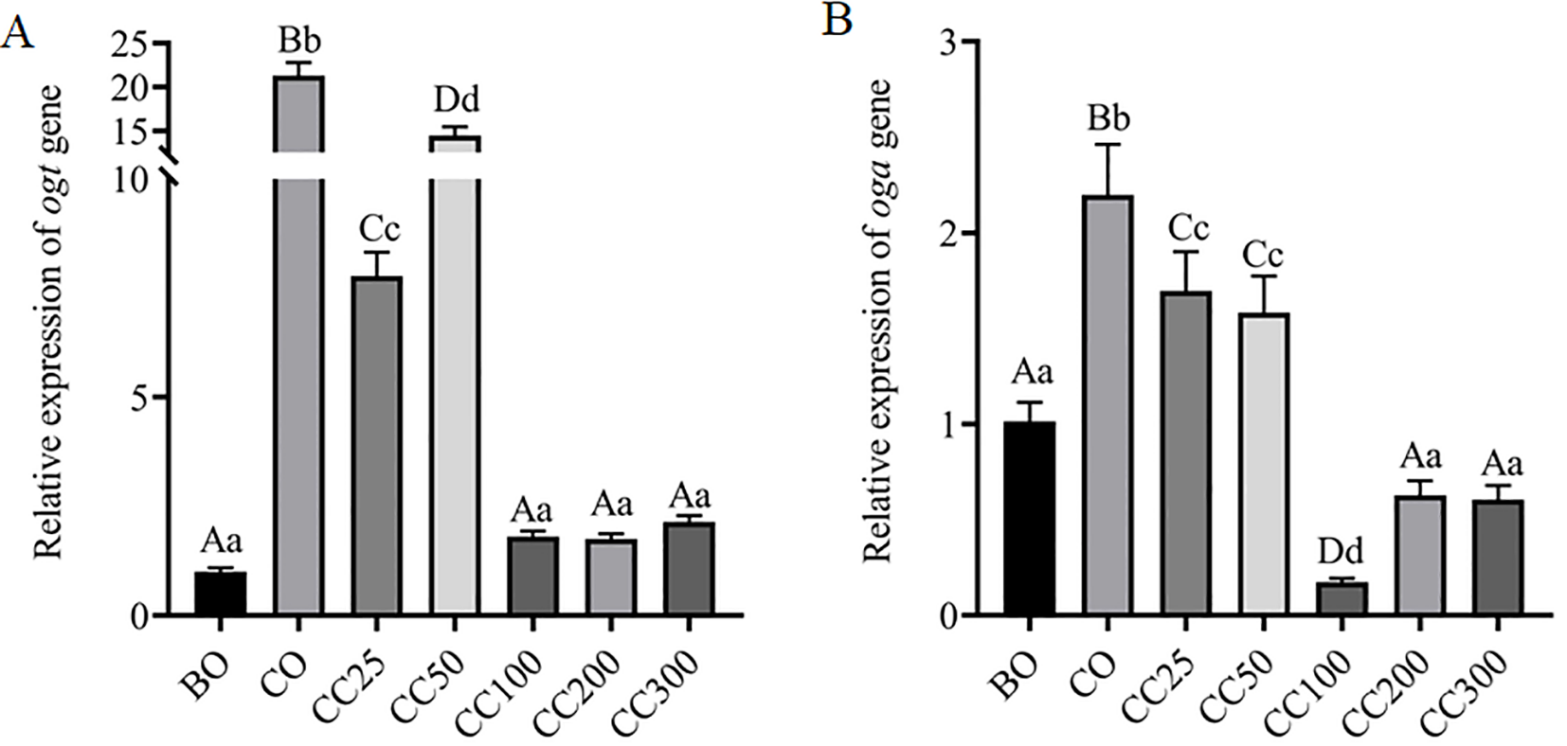
RT-qPCR analysis of OGA **(A)** and OGT **(B)** mRNA expression levels in mouse testes.

**Figure 7:**
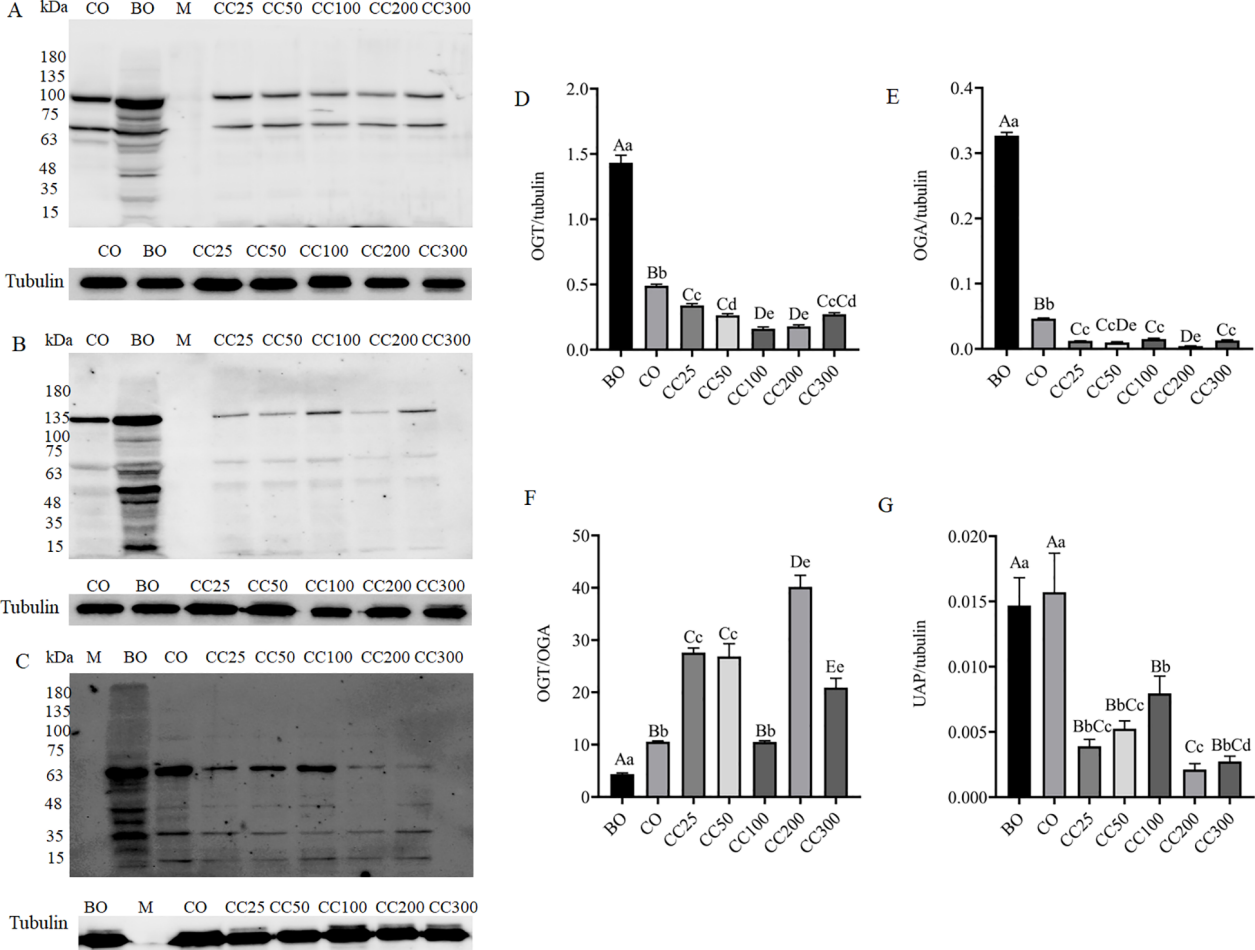
Western blot analysis of the effects of curcumin on the expression of OGT **(A)**, OGA **(B)**, and UAP1 **(C)** in the testes of mice with cryptorchidism. Statistical analysis results of relative expression levels of OGT **(D)**, OGA **(E)**, OGT/OGA **(F)**, and UAP **(G)**. Columns with different uppercase and lowercase letters indicate extremely significant differences (P < 0.01), columns with the same uppercase letters but different lowercase letters indicate significant differences (P < 0.05), and completely identical letters indicate non-significant differences (P > 0.05).

To investigate the effects of curcumin on the expression of proteins related to *O*-GlcNAc glycosylation modification in the testes of mice with cryptorchidism, this study examined the mRNA and protein expression levels of OGT and OGA in mouse testes. Compared to the BO group, the mRNA expression levels of OGT and OGA in the CO group were significantly increased (P < 0.001) (**Figures 6A, B**), but the protein expression levels of OGT (approximately 110 kDa) and OGA (approximately 130 kDa) in the CO group were significantly decreased (P < 0.001) (**Figure 7A, B, D, E**). Meanwhile, compared to the BO group, the OGT/OGA ratio in the CO group was significantly increased (**Figure 7F**).

Compared to the CO group, supplementation with different doses of curcumin further reduced the mRNA (**Figures 6A, B**) and protein expression levels of OGT and OGA (**Figures 7A, B, D, E**). Additionally, the OGT/OGA ratio in the testes of mice supplemented with different doses of curcumin was significantly increased (**Figure 7F**), but there was no significant difference in the OGT/OGA ratio between the CC100 group and the CO group (**Figure 7C**). Next, we continued to explore the expression level of UAP1, a key rate-limiting enzyme in UDP-GlcNAc biosynthesis. Compared to the BO group, there was no significant change in the UAP1 expression level in the CO group (P > 0.05) (**Figure 7G**). However, after supplementation with different doses of curcumin, the expression level of UAP1 was significantly reduced (P < 0.001) (**Figure 7G**), with no significant differences among different doses (P > 0.05, except for the CC200 group) (**Figure 7G**). This study’s quantification relies solely on target band intensities, disregarding protein modifications or degradation effects. Despite our efforts to standardize procedures and reagents in total protein extraction from testis samples across groups, ensuring consistent target band outcomes in replicated experiments, non-specific bands varied. We posit three explanations: heightened non-specific binding in BO controls, elevated target protein modifications yielding multiple band appearances, and reagent batch discrepancies impacting reproducibility.

### 3.6 Molecular docking reveals high-affinity binding of curcumin to OGA and OGT via hydrogen bonding and hydrophobic interactions

**Figure 8:**
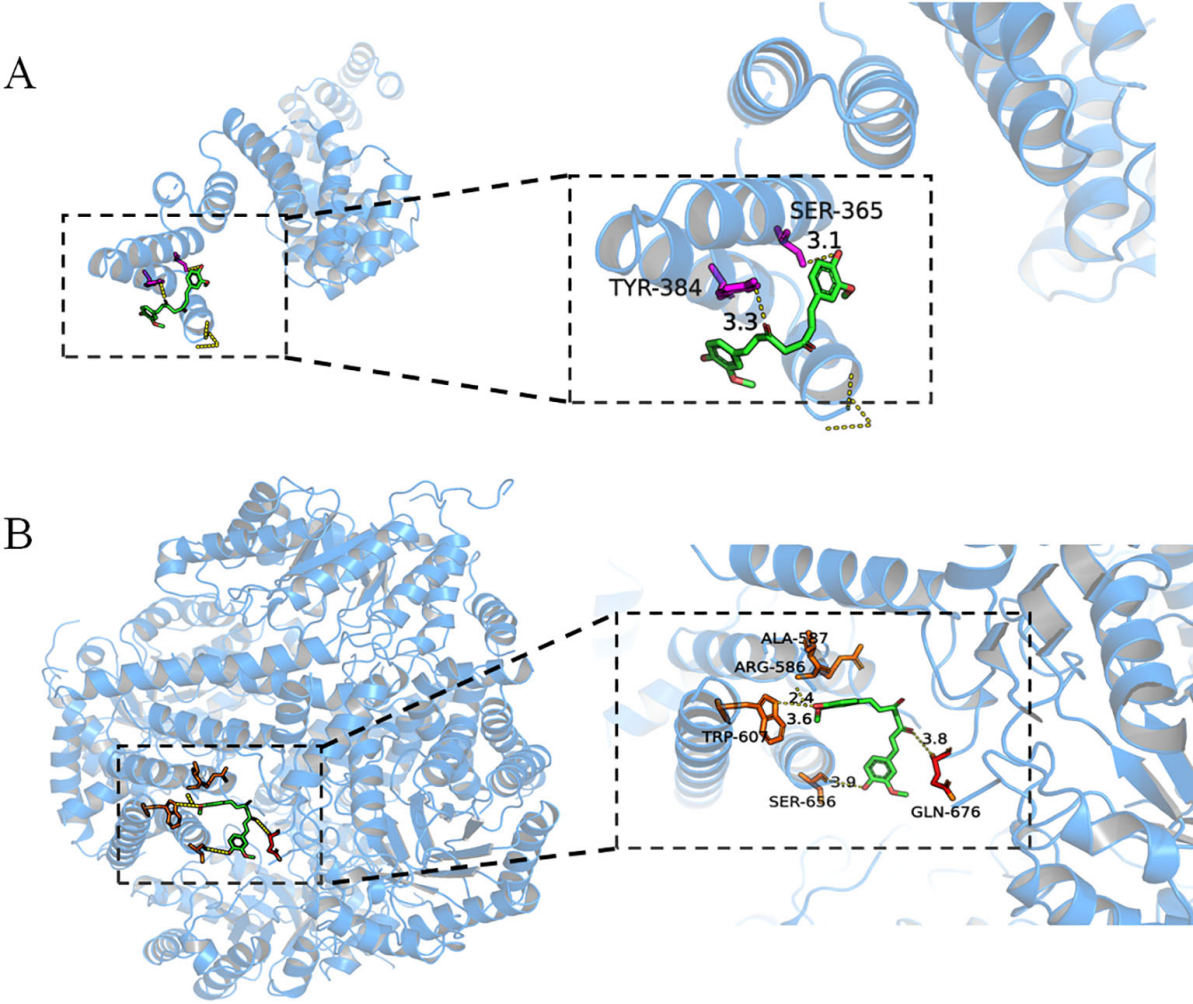
Binding mode of curcumin to OGT **(A)** and OGA **(B)** by molecular docking.

Molecular docking analysis was performed to evaluate the affinity of curcumin for the OGT and OGA. The binding poses and interactions of curcumin with two proteins were obtained with Autodock Vina v.1.2.2 and binding energy for each interaction was generated (**Figure 8**). Results showed that curcumin bound to its protein targets through visible hydrogen bonds interactions. Moreover, the hydrophobic pockets of each targets were occupied successfully by curcumin. OGA and OGT had low binding energy of -7.9 and -6.2 kcal/mol, indicating highly stable binding.

## 4 Discussion

### 4.1 Curcumin exhibits significant protective effects on the reproductive function of mice with cryptorchidism

This study explored the effects of curcumin on the weight, organ coefficients, sperm count and motility, and testicular histology of mice with cryptorchidism by constructing a mouse cryptorchidism model and supplementing it with different doses of curcumin. The results indicated that an appropriate dose of curcumin has a certain protective effect on the reproductive function of mice with cryptorchidism. Notably, the weight of the mice was not significantly affected by cryptorchid surgery. However, curcumin supplementation at appropriate doses increased the weight of the mice (**Table 1**), suggesting that curcumin did not cause toxic damage to mice. Organ coefficients can reflect the effect of drugs on organs, reflecting changes such as atrophy, degenerative changes, congestion, edema, hyperplasia, and hypertrophy of the parenchymal organs (33). The study found that apart from a significant decrease in testicular coefficient, other organ coefficients, such as the brain, cerebellum, heart, kidneys, spleen, stomach, seminal vesicles, lungs, colon, liver, and pancreas, did not change significantly after cryptorchid surgery, indicating that cryptorchid surgery is an ideal method for creating a mouse model of reproductive disorders with low or no toxicity. Meanwhile, the testicular coefficient of mice treated with curcumin increased, with a significant increase in the CC300 group (P < 0.01), suggesting that curcumin may repair testicular damage caused by cryptorchid surgery. Additionally, the liver coefficient was significantly reduced in the CC200 (P < 0.05) and CC300 groups (P < 0.01), indicating that high-dose curcumin may cause liver damage in mice (**Supplementary Figure S1G**). Furthermore, the pancreatic coefficient was significantly reduced in the CC50 and CC300 groups, suggesting that curcumin may affect pancreatic growth and development in mice (**Supplementary Figure S1J**). In summary, the experimental results indicate that curcumin is non-toxic or has a low toxicity profile, and doses of 25 mg/kg, 50 mg/kg, and 100 mg/kg are safe in mice. Although high-dose curcumin can better repair testicular damage, there is a risk of liver damage. This study provides theoretical support for subsequent clinical drug trials.

Additionally, in terms of sperm count, cryptorchid surgery led to stagnation of spermatogenesis in mice, and no sperm was found (**Figure 1**). However, curcumin supplementation significantly promoted spermatogenesis and increased sperm density. Notably, the sperm count in the CC100 group increased the most significantly, approaching the level of the normal group. Testicular pathological analysis (**Figure 2**) revealed that cryptorchid surgery led to significant morphological changes in the testes of mice, including atrophy of the seminiferous tubules and disruption of the spermatogenic epithelium. Curcumin supplementation significantly alleviated these adverse histological changes, resulting in the regular arrangement of germ cells at all levels, smaller cavities, larger lumen diameters, and the presence of mature sperm. However, there was no significant difference between the CC100 group and the BO group.

Simultaneously, there is a complex interaction between hormone levels, antioxidant capacity, and spermatogenesis. Understanding how these hormones affect spermatogenesis is crucial for diagnosing and treating male infertility (34, 35). This study further revealed the important role of the hypothalamic-pituitary-testicular axis in the male reproductive system by analyzing changes in multiple reproduction-related hormone levels and exploring the regulatory effects of curcumin on these hormones. The results showed that cryptorchid surgery significantly affected the levels of key reproductive hormones such as testosterone, follicle-stimulating hormone, luteinizing hormone, total superoxide dismutase, and estradiol in mice. These hormone levels were directly related to spermatogenesis and reproductive health status in mice. As a natural drug with antioxidant and endocrine regulatory effects, curcumin can regulate hormone and antioxidant levels in mice with cryptorchidism, indicating that curcumin may promote spermatogenesis and improve reproductive health by reducing oxidative stress and improving endocrine disorders. Although this study revealed that 100 mg/kg/day curcumin administration increased sperm count and modulated hormonal profiles (e.g., lowering FSH and estradiol more than other curcumin doses), the underlying mechanisms likely involve multifactorial interactions requiring further investigation.

Therefore, this study demonstrated that curcumin doses ranging from 25 to 300 mg/kg promote spermatogenesis and improve testicular histological changes in mice with cryptorchidism, thus exerting protective effects on their reproductive function. Notably, curcumin supplementation at a dose of 100 mg/kg can repair testicular damage caused by cryptorchid surgery, restore spermatogenesis function, and does not cause harm to other organs. This study provides theoretical and experimental support for the application of curcumin in the field of male reproductive health. However, it should be noted that high-dose curcumin may have certain toxic effects on organs such as the liver, so the dose needs to be strictly controlled and its safety needs to be closely monitored in practical applications. Future studies can further explore the specific mechanism by which curcumin regulates reproductive hormone levels and its potential applicability in the clinical treatment of reproductive system diseases.

### 4.2 Curcumin promotes spermatogenesis in mice with cryptorchidism by regulating testicular protein *O*-GlcNAcylation

Spermatogenesis is a multi-stage process involving the transformation of spermatogonia into spermatocytes, haploid spermatids, and then mature sperm. This complex progression is characterized by mitosis, meiosis, and accompanying epigenetic modifications such as histone methylation and acetylation (36). These epigenetic modifications are crucial for ensuring correct chromosome pairing, successful separation of bivalent chromosomes, and timely expression of meiosis-specific genes. Therefore, any factors that affect these key epigenetic modifications may directly impact the normal progress of spermatogenesis. *O*-GlcNAc modification, an important post-translational modification, can regulate various cellular functions, including cell cycle progression, cell proliferation, and differentiation (37, 38). For example, the role of *O*-GlcNAc modification in embryonic development has been extensively studied, with the genetic knockout of OGA leading to embryonic development delay and neonatal death, indicating that OGA plays an indispensable role in embryonic development (37). Additionally, *O*-GlcNAc modification is closely related to neural development, and its decrease during early embryonic development stages is associated with neurodevelopmental disorders (39). Tourzani et al. reported varying degrees of *O*-GlcNAc glycosylation modification in the testes, epididymides, and sperm of mice (40). The Kile research group suggested that spermatogenesis abnormalities caused by reducing UDP-GlcNAc concentrations in mice were likely due to changes in *O*-GlcNAc glycosylation modification levels (41).

Although direct evidence of the specific role of *O*-GlcNAc in spermatogenesis is currently insufficient, its known functions in regulating the cell cycle, cell proliferation, and differentiation, as well as its crucial role in embryonic and neural development, suggest that *O*-GlcNAc modification may indirectly regulate spermatogenesis by affecting protein function and stability in these key processes. In this study, two highly specific commercial antibodies, RL-2 and CTD110.6, were used to detect changes in the level of testicular protein *O*-GlcNAcylation in mice. As shown in **Figure 4**, the results indicated that, compared to the normal control group (BO group), the level of testicular protein *O*-GlcNAcylation in the cryptorchidism model group (CO group) was significantly reduced. This finding is consistent with our previous reports (8) and further confirms the close correlation between spermatogenesis disorders and reduced levels of testicular protein *O*-GlcNAcylation in mice with cryptorchidism. To investigate whether spermatogenesis disorders could be improved by regulating *O*-GlcNAc modification, curcumin supplementation experiments were conducted. The experimental results showed that after supplementation with different doses of curcumin, the level of testicular protein *O*-GlcNAcylation in mice was significantly increased, accompanied by improvements in spermatogenesis, indicating that promoting spermatogenesis in mice with cryptorchidism can be achieved by increasing the level of testicular protein *O*-GlcNAcylation. Notably, in addition to changes in total testicular protein *O*-GlcNAcylation levels accompanying spermatogenesis disorders and repair, this study also observed that testicular proteins of specific molecular weight (approximately 35 kDa, 48 kDa, 100 kDa, and 63 kDa) were closely related to spermatogenesis. These specific proteins may be key proteins involved in spermatogenesis, and their *O*-GlcNAc modification status may directly affect their function and stability, thereby regulating spermatogenesis. Therefore, further research into the role of these specific proteins in spermatogenesis and how *O*-GlcNAc modification affects their function will be important to comprehensively understand the regulatory role of *O*-GlcNAc modification in spermatogenesis. Consistent with previous reports (8, 39), this study once again confirms the relationship between testicular protein *O*-GlcNAcylation levels and spermatogenesis. Specifically, elevated testicular protein *O*-GlcNAcylation levels can restore spermatogenesis in cryptorchid mice, while reduced levels inhibit spermatogenesis. These conclusions offer new perspectives for developing spermatogenic drugs or male contraceptives.

### 4.3 Possible reasons curcumin regulates changes in testicular protein *O*-GlcNAcylation

In the process of testicular protein *O*-GlcNAc glycosylation, OGT transfers GlcNAc from UDP-GlcNAc to the hydroxyl groups of threonine and/or serine residues of target proteins, while OGA hydrolyzes GlcNAc from *O*-GlcNAc-modified proteins. To investigate the effects of curcumin on the expression of proteins related to *O*-GlcNAc glycosylation modification in the testes of cryptorchid mice, this study examined the mRNA and protein expression levels of OGT and OGA in mouse testes. Compared to the normal control group (BO group), the mRNA expression levels of OGT and OGA in the cryptorchid model group (CO group) were significantly increased (P < 0.001) (**Figures 6A, B**). However, despite the significant increase in mRNA levels, the expression levels of OGT protein (approximately 110 kDa) and OGA protein (approximately 130 kDa) in the CO group were significantly decreased (P < 0.001) (**Figures 7A, B, D, E**). This finding suggests that the final protein expression level is not solely determined by mRNA levels but is regulated by multiple factors, including mRNA stability, translation efficiency, and protein degradation rate. For example, previous studies have demonstrated that although α and β cells in the pancreas have high levels of OGT mRNA expression, protein levels are not always directly correlated (42). Combining the results of testicular protein *O*-GlcNAcylation analysis (**Figures 4A–D**), we speculate that cryptorchid surgery may affect the level of testicular protein *O*-GlcNAcylation by reducing the activity or function of OGT and OGA, rather than just their expression levels, leading to spermatogenic disorders. Interestingly, compared to the BO group, the OGT/OGA ratio in the CO group was significantly increased (**Figure 7F**). We speculate that under cryptorchid conditions, the mouse testes attempt to dynamically regulate the *O*-GlcNAcylation levels of specific proteins by adjusting the relative activities of OGT and OGA to cope with the damage caused by surgery. However, the specific mechanism of this regulation needs to be further investigated.

After supplementing with different doses of curcumin, the mRNA and protein expression levels of OGT and OGA were decreased (**Figures 6A, B**; **Figures 7A, B, D, E**). However, this change was not entirely consistent with the changes in testicular protein *O*-GlcNAcylation levels (**Figure 4**). Following curcumin supplementation, the protein expression levels of both OGT and OGA were reduced (**Figures 6**, **7**), yet testicular protein *O*-GlcNAcylation levels were significantly elevated (**Figure 4**). These findings suggest that curcumin likely regulates testicular protein *O*-GlcNAcylation by modulating the enzymatic activities of OGT and OGA rather than their expression levels. Molecular docking analysis revealed high-affinity binding of curcumin to both OGA and OGT, with a stronger affinity for OGA, indicating its potential to directly target these enzymes and influence the dynamic regulation of protein *O*-GlcNAcylation (**Figure 8**). We hypothesize that curcumin’s higher affinity for OGA may preferentially inhibit its hydrolytic activity, leading to intracellular accumulation of *O*-GlcNAcylation, while its moderate binding to OGT may partially restrict excessive *O*-GlcNAcylation, thereby restoring *O*-GlcNAc homeostasis. In other words, curcumin may recalibrate *O*-GlcNAcylation equilibrium by adjusting the relative enzymatic activities of OGT and OGA rather than their absolute expression levels. However, the current static docking results require validation through molecular dynamics simulations to assess binding conformation stability, complemented by *in vitro* enzymatic activity assays to confirm functional effects.

In addition, the concentration of UDP-GlcNAc is a key factor in regulating *O*-GlcNAcylation (43). Cryptorchid surgery may reduce the concentration of UDP-GlcNAc, thereby limiting the availability of substrates for *O*-GlcNAc modification and affecting the level of testicular protein *O*-GlcNAcylation. A decrease in *O*-GlcNAcylation levels may directly interfere with the function and stability of proteins related to spermatogenesis, ultimately leading to spermatogenic disorders. Notably, after curcumin supplementation, the UDP-GlcNAc concentrations in mouse testicular tissue and serum were significantly increased (**Figures 5A, B**). This result suggests that curcumin may promote UDP-GlcNAc biosynthesis or reduce its consumption through some mechanism, thereby increasing intracellular UDP-GlcNAc concentrations. The UDP-GlcNAc concentration increased with increasing levels of testicular protein *O*-GlcNAcylation, potentially helping restore the normal function and stability of proteins related to spermatogenesis, thereby promoting sperm production. These findings suggest that the concentration of UDP-GlcNAc plays an important role in spermatogenesis. Supplementing UDP-GlcNAc or promoting its accumulation within cells may be a potential strategy for treating cryptorchidism and its resulting spermatogenic disorders. Future studies should investigate how curcumin affects UDP-GlcNAc biosynthesis and consumption pathways, as well as the specific role of UDP-GlcNAc in spermatogenesis, to provide insights into new strategies and methods for improving reproductive health. Our results demonstrate that curcumin promotes spermatogenesis by regulating testicular protein *O*-GlcNAcylation levels, a process associated with the expression of OGT and OGA, as well as the concentration of UDP-GlcNAc. Integrated molecular docking and UDP-GlcNAc analyses suggest curcumin may restore *O*-GlcNAc homeostasis through a dual action: inhibiting OGA hydrolysis via high-affinity binding and enhancing OGT activity via UDP-GlcNAc accumulation, likely synergistically restoring *O*-GlcNAc equilibrium. However, this study did not examine the metabolites of curcumin *in vivo* or their mechanisms of action, necessitating further investigation.

## 5 Conclusion

This study explored the positive effects of curcumin on spermatogenesis in mice with cryptorchidism and its potential mechanisms. The experimental results demonstrated that curcumin can effectively alleviate reproductive function damage caused by cryptorchidism, especially at an optimum dose of 100 mg/kg, significantly improving testicular coefficients, sperm counts, testicular tissue morphology, and hormone levels in mice. Further mechanistic studies revealed that curcumin promotes sperm production by increasing the level of testicular protein *O*-GlcNAcylation, specifically by increasing UDP-GlcNAc concentration and regulating OGT/OGA expression. Molecular docking and UDP-GlcNAc analyses further suggest this occurs through dual mechanisms: inhibiting OGA via high-affinity binding and enhancing OGT activity through substrate accumulation, synergistically rebalancing *O*-GlcNAc glycosylation dynamics. This study not only provides scientific evidence for the application of curcumin in male reproductive health but also reveals the important role of *O*-GlcNAcylation modification in the process of spermatogenesis. We expect curcumin to become a promising candidate drug for adjuvant treatment of male infertility, bringing new hope to numerous patients.

## Data Availability

The original contributions presented in the study are included in the article/**Supplementary Material**. Further inquiries can be directed to the corresponding authors.
