# Supplementary material for: Curcumin promotes spermatogenesis in mice with cryptorchidism by regulating testicular protein O-GlcNAcylation
Source: Front Endocrinol (Lausanne). 2025 Jun 30;16:1555721. doi: 10.3389/fendo.2025.1555721 (PMC12256208; doi:10.3389/fendo.2025.1555721)
Supplement: Supplementary file 1 [file SupplementaryFile1.docx]

Supplemental materials

Genomic infrastructure for cetacean research and conservation: Reference genomes for eight families spanning the cetacean tree of life

**Contents**

[Supplemental methods: 2](#_Toc187840783)

[Table S1. Pairwise genome alignment files 4](#_Toc187840784)

[Table S2. Mitogenome accession IDs 5](#_Toc187840785)

[Table S3. SRA and BioSample information 6](#_Toc187840786)

[Table S4. Generation times 8](#_Toc187840787)

[Table S5. Pygmy sperm whale genome annotation comparison 9](#_Toc187840788)

[Table S6. Runs of Homozygosity 10](#_Toc187840789)

[Table S7. ROH size ranges 12](#_Toc187840790)

[Table S8: Length of MCH region blocks 13](#_Toc187840791)

[Table S9. *IGF1* sites potentially associated with body size 14](#_Toc187840792)

[Table S10. *IGF1* site SNV positions and nucleotides 15](#_Toc187840793)

[Table S11. VCF file information 16](#_Toc187840794)

[Figure S1. Consensus nuclear locus phylogeny 17](#_Toc187840795)

[Figure S2. Ancestral linkage group karyotypes 18](#_Toc187840796)

[Figure S3. Genome annotation summary 23](#_Toc187840797)

[Figure S4. Repeat landscape profile comparison 24](#_Toc187840798)

[Figure S5. Heterozygosity per 1MB window 25](#_Toc187840799)

[Figure S6. Genome coverage comparison 29](#_Toc187840800)

[Figure S7. Historical demography plots 32](#_Toc187840801)

[Figure S8: MHC class I and class IIa assembly improvements 37](#_Toc187840802)

[Figure S9. IGF1 gene type 1 SNV correlation with body size 38](#_Toc187840803)

[Figure S10. IGF1 gene type 2 SNV correlation with body size 38](#_Toc187840804)

[Figure S11: The MHC region from GABBR1 gene to ELOVL5 39](#_Toc187840805)

[References 44](#_Toc187840806)

# Supplemental methods:

Sequencing and assembly of *Inia geoffrensis*

We obtained a blood sample from an Amazon river dolphin (ID “Orinoco”) that was captured in Rio Negro, Venezuela, in 1973 and then brought to Zoo Duisburg in Germany, where he lived until his death in 2020. The blood sample was collected during routine veterinary care, transferred to an EDTA-coated tube, incubated for about 10 minutes at room temperature and then stored on dry ice. For the lysis of the blood sample we used the Red Blood Cell (RBC) lysis buffer from Qiagen (Hilden, Germany) and then proceeded with the DNA extraction protocol according to Sambrook and Russell (2001). DNA concentration and DNA fragment length were assessed using the Qubit dsDNA BR Assay kit on the Qubit Fluorometer (Thermo Fisher Scientific, Waltham, USA) and the Genomic DNA Screen Tape on the Agilent 2200 TapeStation system (Agilent Technologies), respectively.

Three SMRTbell libraries were prepared according to the SMRTbell Express Prep Kit v2.0 instructions (Pacific Biosciences, Menlo Park, CA). Total input DNA was approximately 2-3 µg per library. Two of the prepared libraries were loaded twice on the Pacbio sequencing machine. A total of five SMRT cell sequencing runs were performed in CCS mode on the Sequel System IIe using the Sequel II Binding Kit 2.2 (Pacific Biosciences). Adaptive loading was used to load libraries at an on-plate concentration of 80 pM.

In total five SMRT cells were sequenced on PacBio Sequel IIe instruments. The first three SMRT cells were sequenced without on-board calling of HiFi reads, whereas for the last two SMRT cells this option was enabled. HiFi calling from subreads was conducted with PacBio’s tool ccs 4.2.0-1 (<https://github.com/PacificBiosciences/ccs>). HiFi reads from on-board calling were extracted from the bam file with samtools and a custom perl script if rq was greater or equal 0.99.

The Omni-C library was prepared from 1 ml of blood from the same individual using the Dovetail® Omni-C® Kit (Cantata Bio LLC, Scotts Valley, CA) according to the manufacturer’s protocol version 1.2. After proximity ligation, the DNA was converted into a sequencing library using Illumina-compatible adapters. Fragment size distribution and concentration of the Omni-C library were assessed using the Agilent 2200 TapeStation system and the Qubit Fluorometer and Qubit dsDNA HS reagents Assay kit (Thermo Fisher Scientific), respectively. The library, with an insert size of 350 bp, was sequenced on the NovaSeq 6000 platform at Novogene (UK) using a 150 paired-end sequencing strategy, resulting in an output of 150 Gb.

Three different tissue samples preserved in RNAlater for RNA extraction were obtained from the Biobank of The Leibniz Institute for the Analysis of Biodiversity Change in Bonn, Germany. Total RNA was extracted from liver (Biobank Tissue reference number: ZFMK-TIS-55018), spleen (Biobank Tissue reference number: ZFMK-TIS-55034) and testis (Biobank Tissue reference number: ZFMK-TIS-55039) from the same individual using TRIzol reagent (Invitrogen) according to the manufacturer's instructions. The quality and concentration of each extraction was assessed using the TapeStation 2200 (Agilent Technologies) and the Qubit Fluorometer with the RNA BR Reagents Assay Kit (Thermo Fisher Scientific, Waltham, MA). The RNA extractions were then sent to Novogene (UK) for Illumina paired-end 150 bp RNA-seq of a cDNA library (insert size: 350 bp) with an expected output of 9 Gb (liver and spleen) and 18 Gb (testis).

Contig assembly was conducted with hifiasm 0.16.1 (Cheng et al., 2021; Cheng et al., 2022) using the HiC data and HiFi reads from the five SMRT cells as input. To reduce haplotypic duplication, purge_dups 1.2.5 (Guan et al., 2020) was executed on contigs of haplotype 1 and 2 separately, following the recommended workflow. Cutoffs for purge_dups were manually adjusted to 5,15,25,23,50,65 and 5,15,25,21,50,70 for haplotype 1 and 2 respectively. Subsequently the assembly was polished using a workflow including DeepVariant (v1.2; Poplin et al., 2018). First, the HiFi reads used for assembly were mapped against the contigs of haplotype 1 and 2 separately with minimap2 2.24 (Li, 2018; 2021) and the options “-a -x map-hifi”. The bam file was sorted by coordinate with samtools 1.15 (Danecek et al., 2021) and duplicated HiFi reads were removed with Picard (v2.26.10; Broad Institute, http://broadinstitute.github.io/picard/) MarkDuplicates and the option --REMOVE_DUPLICATES. The assembly fasta and filtered bam files were indexed with samtools faidx and index commands, respectively. DeepVariant 1.2 was used to call SNPs. To keep only homozygous variants, SNPs were subsequently filtered using bcftools view 1.15 (Danecek et al., 2021) with the options -f 'PASS' -i 'GT="1/1"' . Then, the vcf file containing the homozygous SNPs was indexed with tabix from htslib 1.15 (Bonfield et al., 2021) to finally apply the variants in the filtered assemblies with bcftools consensus. Purged and polished contigs of both haplotypes were scaffolded with YaHS 1.1 (Zhou et al., 2023). To do so, HiC reads were first mapped with the Arima mapping pipeline (https://github.com/VGP/vgp-assembly/blob/master/pipeline/salsa/arima_mapping_pipeline.sh), in combination with bwa mem 0.7.17 (Li, 2013), samtools 1.15, picard 2.26.10, and java 1.8.0 (Arnold et al., 2005). Afterwards, the bam file was processed along with the contigs in YaHS. Visualization and manual curation from scaffolds of both haplotypes separately was conducted with rapid curation (<https://gitlab.com/wtsi-grit/rapid-curation>), following the suggested workflow.

# Table S1. Pairwise genome alignment files

List of pairwise genome alignment files used for synteny and pairwise divergence analyses. All pairwise statistics are based on alignment blocks ≥1000bp. NOTE: the primary haplotypes listed in Table 1 of the paper were used for all species except. G. melas and D. delphis. For those, two species, the primary haplotype was updated to fix assembly errors detected during our MHC analysis, and the alignments were re-run using the updated primary haplotype assemblies (mDelDel1.2, GCA_949987515.2; mGloMel1.2, GCA_963455315.2).

| Species 1 | Species 2 | Filename | Average percent pairwise divergence | Number of blocks >1kb | Average length of blocks (>1kb) |
| --- | --- | --- | --- | --- | --- |
| B. acutorostrata | E. glacialis | BAC-EGL.1-1.maf.gz | 1.97 | 248599 | 1592 |
| B. acutorostrata | E. robustus | BAC-ERO.1-1.maf.gz | 1.45 | 252655 | 1593 |
| B. musculus | B. ricei | BMU-BRI.1-1.maf.gz | 1.03 | 259329 | 1605 |
| B. musculus | E. robustus | BMU-ERO.1-1.maf.gz | 1.25 | 257047 | 1600 |
| D. delphis | S. coeruleoalba | DDE-SCO.1-1.maf.gz | 0.44 | 322137 | 1686 |
| D. delphis | T. truncatus | DDE-TTR.1-1.maf.gz | 0.44 | 322281 | 1678 |
| G. melas | O. orca | GME-OOR.1-1.maf.gz | 1.11 | 325815 | 1689 |
| H. ampullatus | I. geoffrensis | HAM-IGE.1-1.maf.gz | 3.75 | 227226 | 1578 |
| H. ampullatus | M. densirostris | HAM-MDE.1-1.maf.gz | 1.34 | 244091 | 1580 |
| Hippo | E. glacialis | HipAM-EGL.1-1.maf.gz | 8.58 | 24550 | 1647 |
| K. breviceps | B. ricei | KBR-BRI.1-1.maf.gz | 4.12 | 204608 | 1539 |
| K. breviceps | M. densirostris | KBR-MDE.1-1.maf.gz | 4.30 | 193228 | 1524 |
| L. albirostris | O. orca | LAL-OOR.1-1.maf.gz | 1.08 | 260997 | 1603 |
| L. albirostris | S. coeruleoalba | LAL-SCO.1-1.maf.gz | 1.13 | 258491 | 1599 |
| N. asiaeorientalis | I. geoffrensis | NAS-IGE.1-1.maf.gz | 3.30 | 244922 | 1592 |
| N. asiaeorientalis | P. sinus | NAS-PSI.1-1.maf.gz | 0.52 | 265853 | 1604 |
| P. phocoena | P. sinus | PPH-PSI.1-1.maf.gz | 0.41 | 268332 | 1607 |
| P. phocoena | T. truncatus | PPH-TTR.1-1.maf.gz | 2.24 | 249503 | 1594 |

# Table S2. Mitogenome accession IDs

Mitochondrial genome accession ID’s for sequences used to map mitochondrial reads, and for phylogenetic analysis. Short read data for each species was mapped to a reference mitochondrial genome from the same species, except for the harbor porpoise, which was mapped to a vaquita mitochondrial genome sequence. The Rice’s whale genome was assembled de novo from whole genome sequence data (SRR10331559) using NovoPlasty v4.1 (Dierckxsens et al., 2017).

| Common name | Latin name | Mitogenome accession |
| --- | --- | --- |
| Common minke whale | *Balaenoptera acutorostrata* | AP006468 |
| Blue whale | *Balaenoptera musculus* | MF409242 |
| Rice's whale | *Balaenoptera ricei* | (de novo) |
| Common dolphin | *Delphinus delphis* | OX465349 |
| Gray whale | *Eschrichtius robustus* | NC_005270 |
| North Atlantic right whale | *Eubalaena glacialis* | NC_037444 |
| Long-finned pilot whale | *Globicephala melas* | NC_019441 |
| Northern bottlenose whale | *Hyperoodon ampullatus* | NC_005273 |
| Amazon River dolphin | *Inia geoffrensis* | AJ554059 |
| Pygmy sperm whale | *Kogia breviceps* | NC_005272 |
| White-beaked dolphin | *Lagenorhynchus albirostris* | NC_005278 |
| Blainville's beaked whale | *Mesoplodon densirostris* | KF032860 |
| Yangtze finless porpoise | *Neophocaena asiaeorientalis* | NC_026456 |
| Killer whale | *Orcinus orca* | NC_064558 |
| Harbor porpoise | *Phocoena phocoena* | CM018178.1 |
| Vaquita | *Phocoena sinus* | CM018178.1 |
| Striped dolphin | *Stenella coeruleoalba* | NC_012053 |
| Common bottlenose dolphin | *Tursiops truncatus* | NC_012059 |

# Table S3. SRA and BioSample information

Short-read data for each species mapped to reference genomes. Files starting with SRR were downloaded from NCBI. Files starting with ERR were downloaded from the ENA. Data types: WGS, whole genome shotgun; Hi-C, chromatin cross-linked genome data. Arima, Hi-C data from Arima Genomics. *Data from the same individual as used for reference genome assembly.

| Common name | Species | SRA files | BioSample | Data type | Depth of coverage |
| --- | --- | --- | --- | --- | --- |
| Minke whale | *Balaenoptera acutorostrata* | ERR11040176 | [SAMEA111380540](https://www.ncbi.nlm.nih.gov/biosample/SAMEA111380540/)* | Arima Hi-C | 89 |
| Blue Whale | *Balaenoptera musculus* | SRR5665644 | [SAMN07201754](https://www.ncbi.nlm.nih.gov/biosample/SAMN07201754) | Illumina HiSeq 2000, WGS | 34 |
| Rice's Whale | *Balaenoptera ricei* | SRR10331559 | [SAMN13072001](https://www.ncbi.nlm.nih.gov/biosample/SAMN13072001) | Illumina NovaSeq 6000, WGS | 43 |
| Short-beaked common dolphin | *Delphinus delphis* | ERR11040185 | [SAMEA111380534](https://www.ncbi.nlm.nih.gov/biosample/SAMEA111380534/)* | Arima Hi-C | 94 |
| Gray whale | *Eschrichtius robustus* | [SRR12437599](https://trace.ncbi.nlm.nih.gov/Traces?run=SRR12437599) | [SAMN15801458](https://www.ncbi.nlm.nih.gov/biosample/SAMN15801458) | Illumina NovaSeq 6000, WGS | 40 |
| North Atlantic right whale | *Eubalaena glacialis* | SRR11097130 | [SAMN14122067](https://www.ncbi.nlm.nih.gov/biosample/SAMN14122067) | Illumina NovaSeq 6000, WGS | 35 |
| Long-finned pilot whale | *Globicephala melas* | ERR11837528 | [SAMEA111380538](https://www.ncbi.nlm.nih.gov/biosample/SAMEA111380538/)* | Arima Hi-C | 34 |
| Northern bottlenose whale | *Hyperoodon ampullatus* | ERR10908646 | [SAMEA10839125](https://www.ncbi.nlm.nih.gov/biosample/SAMEA10839125/)* | Arima Hi-C | 76 |
| Amazon River dolphin | *Inia geoffrensis* | [SRR11430609](https://trace.ncbi.nlm.nih.gov/Traces?run=SRR11430609) | SAMN07678125 | Illumina HiSeq 2500*, WGS* | 25 |
| Pygmy Sperm Whale | *Kogia breviceps* | SRR11430611 | [SAMN07678126](https://www.ncbi.nlm.nih.gov/biosample/SAMN07678126) | Illumina HiSeq 2500*, WGS* | 30 |
| White-beaked dolphin | *Lagenorhynchus albirostris* | ERR11042965 | SAMEA111380547* | Arima Hi-C | 62 |
| Blainville's beaked whale | *Mesoplodon densirostris* | SRR13167975 | [SAMN16895766](https://www.ncbi.nlm.nih.gov/biosample/SAMN16895766) | Illumina NovaSeq 6000, WGS | 39 |
| East Asian finless porpoise | *Neophocaena asiaeorientalis sunameri* | [SRR21047154](https://trace.ncbi.nlm.nih.gov/Traces?run=SRR21047154) | SAMN30090519* | MGISEQ-2000RS | 33 |
| Killer Whale | *Orcinus orca* | [SRR574970, SRR574978, SRR574980, SRR574981](https://trace.ncbi.nlm.nih.gov/Traces?run=SRR574978) | SAMN01180276* | Illumina HiSeq 2000, WGS | 26 |
| Harbor porpoise | *Phocoena phocoena* | SRR11432011 | SAMN07678060 | Illumina HiSeq 2500, WGS | 41 |
| Vaquita | *Phocoena sinus* | SRR15435906 | [SAMN20563478](https://www.ncbi.nlm.nih.gov/biosample/SAMN20563478) | HiSeq X Ten, WGS | 38 |
| Striped dolphin | *Stenella coeruleoalba* | ERR11042965 | [SAMEA111380539](https://www.ncbi.nlm.nih.gov/biosample/SAMEA111380539/)* | Arima Hi-C | 87 |
| Common Bottlenose Dolphin | *Tursiops truncatus* | https://genomeark.s3.amazonaws.com/index.html?prefix=species/Tursiops_truncatus/mTurTru3/genomic_data/illumina/ | SAMN12611942 | Illumina HiSeq | 71 |

# Table S4. Generation times

Generation times (T(r=0) from Taylor et al. (2007)), except for vaquita and Blainville's beaked whale, which were estimated based on congeneric species.

| Common name | Species name | Generation time |
| --- | --- | --- |
| Minke whale | *Balaenoptera acutorostrata* | 22.1 |
| blue whale | *Balaenoptera musculus* | 30.8 |
| Rice's Whale | *Balaenoptera ricei* | 18.4 |
| common dolphin | *Delphinus delphis* | 14.8 |
| gray whale | *Eschrichtius robustus* | 22.9 |
| north Atlantic right whale | *Eubalaena glacialis* | 35.7 |
| long-finned pilot whale | *Globicephala melas* | 24.0 |
| northern bottlenose whale | *Hyperoodon ampullatus* | 17.8 |
| Amazon River dolphin | *Inia geoffrensis* | 10.2 |
| pygmy Sperm Whale | *Kogia breviceps* | 12.1 |
| white-beaked dolphin | *Lagenorhynchus albirostris* | 18.1 |
| Blainville's beaked whale | *Mesoplodon densirostris* | 20.0 |
| Yangtze finless porpoise | *Neophocaena asiaeorientalis sunameri* | 16.5 |
| killer whale | *Orcinus orca* | 25.7 |
| harbor porpoise | *Phocoena phocoena* | 11.9 |
| vaquita | *Phocoena sinus* | 11.9 |
| striped dolphin | *Stenella coeruleoalba* | 22.5 |
| common bottlenose dolphin | *Tursiops truncatus* | 21.1 |

# Table S5. Pygmy sperm whale genome annotation comparison

Changes in annotation for the pygmy sperm whale (*Kogia breviceps*) haplotype 1 after initial annotation based on transcriptomic data from the sperm whale (*Physeter catadon*), and a second annotation based on transcriptomic data (RNAseq and IsoSeq, multiple tissues) from the pygmy sperm whale. Raw data from NCBI annotation report release GCF_026419965.1-RS_2024_07 (https://www.ncbi.nlm.nih.gov/refseq/annotation_euk/Kogia_breviceps/GCF_026419965.1-RS_2024_07/)

| Gene Category | count | % of all* | % present in both releases* | |
| --- | --- | --- | --- | --- |
| identical | 5349 | 5.3% | 19% |  |
| minor changes | 63212 | 62.9% | 50% |  |
| major changes | 23748 | 23.6% | 20% |  |
| new | 4258 | 4.2% | 11% |  |
| deprecated | 3321 | 3.3% | 9% |  |
| other | 400 | 0.4% | <1% |  |
| sum | 100288 |  |  |  |
| (not counted) | 209 |  |  |  |

* Categories from genes identified in either release (“all”) and in both release annotations. Only categories of genes identified in both annotation releases were reported in the NCBI annotation web report summary.

# Table S6. Runs of Homozygosity

Summary values for heterozygosity and runs of homozygosity. All values are from DeepVariant analyses (see methods) except the heterozygosity value from ANGSD analysis (Het./bp (ANGSD). The heterozygosity values calculated by DeepVariant and ANGSD differ to varying degrees due to the GenotypeQuality filter in DeepVariant, which filtered out a higher proportion of biallelic SNPs in some samples than others. Het./bp = heterozygosity per nucleotide. Het. inside ROH = Heterozygosity/10kb within runs of homozygosity. Het. outside ROH = Heterozygosity/10kb outside of runs of homozygosity. ROH number is the count of runs of homozygosity >100kb. ROH length is the sum of all ROH greater than 100kb. Percent of genome is the ratio of ROH length to genome length (autosomes only).

| **Common name** | **Species** | **Sample data** | **Het./bp (ANGSD)** | **Het./bp (DV)** | **Het. inside ROH** | **Het. outside ROH** | **ROH number** | **ROH length (bp)** | **Percent of genome** | **Genome (bp, autosomes)** |
| --- | --- | --- | --- | --- | --- | --- | --- | --- | --- | --- |
| Common minke whale | *Balaenoptera acutorostrata* | ERR11040176 | 0.00046 | 0.00014 | 0.17 | 1.60 | 2141 | 328,050,000 | 13.93 | 2,354,434,377 |
| Blue whale | *Balaenoptera musculus* | SRR5665644 | 0.00195 | 0.00216 | 2.36 | 24.96 | 1652 | 322,840,000 | 14.42 | 2,239,549,461 |
| Rice's whale | *Balaenoptera ricei* | SRR10331559 | 0.00015 | 0.00014 | 0.07 | 7.11 | 1095 | 1,920,820,000 | 80.08 | 2,398,479,940 |
| Common dolphin | *Delphinus delphis* | ERR11040185 | 0.00172 | 0.00041 | 0.58 | 4.28 | 507 | 76,490,000 | 3.42 | 2,233,809,055 |
| Gray whale | *Eschrichtius robustus* | SRR12437599 | 0.00042 | 0.00042 | 0.39 | 5.14 | 1362 | 427,220,000 | 17.08 | 2,501,546,185 |
| North Atlantic right whale | *Eubalaena glacialis* | SRR11097130 | 0.00031 | 0.00028 | 0.34 | 4.33 | 2074 | 912,890,000 | 36.34 | 2,512,368,044 |
| Long-finned pilot whale | *Globicephala melas* | ERR11837528 | 0.00066 | 0.00039 | 0.26 | 5.66 | 446 | 60,630,000 | 2.72 | 2,231,422,924 |
| Northern bottlenose whale | *Hyperoodon ampullatus* | ERR10908646 | 0.00035 | 0.00013 | 0.17 | 1.62 | 1971 | 392,530,000 | 17.12 | 2,293,183,029 |
| Amazon River dolphin | *Inia geoffrensis* | SRR11430609 | 0.00052 | 0.00047 | 0.46 | 5.77 | 1583 | 344,310,000 | 14.20 | 2,424,521,462 |
| Pygmy sperm whale | *Kogia breviceps* | SRR11430611 | 0.00506 | 0.00552 | 6.28 | 56.93 | 274 | 55,070,000 | 2.34 | 2,352,146,689 |
| White-beaked dolphin | *Lagenorhynchus albirostris* | ERR11042964 | 0.00027 | 0.00005 | 0.00 | 0.75 | 3230 | 687,920,000 | 30.29 | 2,271,262,980 |
| Blainville's beaked whale | *Mesoplodon densirostris* | SRR13167975 | 0.00133 | 0.00143 | 1.33 | 17.32 | 1468 | 387,430,000 | 16.11 | 2,405,383,248 |
| East Asian finless porpoise | *Neophocaena asiaeorientalis sunameri* | SRR21047154 | 0.00110 | 0.00109 | 1.27 | 11.91 | 969 | 196,920,000 | 8.34 | 2,361,171,653 |
| Killer whale | *Orcinus orca* | SRR574970-98 | 0.00039 | 0.00033 | 0.18 | 5.00 | 1942 | 783,460,000 | 33.34 | 2,349,732,658 |
| Harbor porpoise | *Phocoena phocoena* | SRR11432011 | 0.00134 | 0.00151 | 1.68 | 15.73 | 448 | 91,530,000 | 4.08 | 2,242,038,944 |
| Vaquita | *Phocoena sinus* | SRR15435906 | 0.00011 | 0.00009 | 0.11 | 1.16 | 2554 | 554,590,000 | 24.78 | 2,237,877,686 |
| Striped dolphin | *Stenella coeruleoalba* | ERR11042965 | 0.00333 | 0.00095 | 1.31 | 9.51 | 109 | 14,250,000 | 0.63 | 2,277,154,286 |
| Common bottlenose dolphin | *Tursiops truncatus* | pat18819988 | 0.00079 | 0.00082 | 0.93 | 10.79 | 2543 | 565,620,000 | 25.63 | 2,207,150,749 |

# Table S7. ROH size ranges

Count of ROH in three size categories.

| **Common name** | **Species** | **ROH number < 1Mb** | **ROH number > 1Mb** | **ROH number > 5Mb** |
| --- | --- | --- | --- | --- |
| Common minke whale | *Balaenoptera acutorostrata* | 2,138 | 2 | 0 |
| Blue whale | *Balaenoptera musculus* | 1,626 | 23 | 0 |
| Rice's whale | *Balaenoptera ricei* | 633 | 440 | 109 |
| Common dolphin | *Delphinus delphis* | 505 | 2 | 0 |
| Gray whale | *Eschrichtius robustus* | 1,305 | 56 | 5 |
| North Atlantic right whale | *Eubalaena glacialis* | 1,964 | 104 | 30 |
| Long-finned pilot whale | *Globicephala melas* | 446 | 0 | 0 |
| Northern bottlenose whale | *Hyperoodon ampullatus* | 1,946 | 25 | 0 |
| Amazon River dolphin | *Inia geoffrensis* | 1,549 | 28 | 0 |
| Pygmy sperm whale | *Kogia breviceps* | 270 | 3 | 0 |
| White-beaked dolphin | *Lagenorhynchus albirostris* | 3,193 | 31 | 0 |
| Blainville's beaked whale | *Mesoplodon densirostris* | 1,413 | 41 | 3 |
| East Asian finless porpoise | *Neophocaena asiaeorientalis sunameri* | 947 | 21 | 1 |
| Killer whale | *Orcinus orca* | 1,776 | 137 | 6 |
| Harbor porpoise | *Phocoena phocoena* | 439 | 7 | 0 |
| Vaquita | *Phocoena sinus* | 2,519 | 27 | 0 |
| Striped dolphin | *Stenella coeruleoalba* | 109 | 0 | 0 |
| Common bottlenose dolphin | *Tursiops truncatus* | 2,490 | 45 | 0 |

# Table S8: Length of MCH region blocks

Length of MHC region blocks in kb for draft (from DNAzoo, when available) and reference assemblies. See Figure 9 for description of block framework genes. Length includes framework genes. Mfg = missing framework genes, hence length could not be calculated. Na = length not available. ^1^inclomplete NOTCH4 gene.

|  | length of draft assemblies | | | | |  | length of platinum assemblies | | | | | |
| --- | --- | --- | --- | --- | --- | --- | --- | --- | --- | --- | --- | --- |
|  | **ĸ** | **β** | **III** | **III*** | **IIa** |  | **ĸ** | **β** | **III** | **III*** | **IIa** |  |
| **E. glacialis** | **mfg** | **83** | **508** | **209** | **300** |  | **292** | **106** | **544** | **224** | **369** |  |
| **B. acutorostrata** | **mfg** | **124** | **587** | **96** | **324** |  | **286** | **120** | **566** | **97** | **353** |  |
| **E. robustus** | **196** | **70** | **497** | **206** | **341** |  | **287** | **110** | **598** | **213** | **363** |  |
| B. musculus |  |  |  |  |  |  | **257** | **115** | **578** | **217** | **359** |  |
| **B. ricei** | **162** | **60** | **508** | **205** | **311** |  | **285** | **112** | **545** | **214** | **397** |  |
| **K. breviceps** | **173** | **mfg** | **mfg** | **mfg** | **277** |  | **198** | **55** | **543** | **66** | **317** |  |
| H. ampullatus |  |  |  |  |  |  | **211** | **69** | **577** | **198** | **327** |  |
| **M. densirostris** | **125** | **48** | **441** | **199** | **302** |  | **252** | **64** | **549** | **204** | **320** |  |
| I. geoffrensis |  |  |  |  |  |  | **181** | **81** | **541** | **554** | **323** |  |
| N. asiaeorientalis |  |  |  |  |  |  | **285** | **81** | **548** | **658** | **308** |  |
| P. sinus |  |  |  |  |  |  | **201** | **82** | **546** | **552** | **309** |  |
| P. phoceana |  |  |  |  |  |  | **242** | **82** | **547** | **1,095** | **308** |  |
| T. truncatus |  |  |  |  |  |  | **289** | **81** | **546** | **252** | **302** |  |
| D. delphis |  |  |  |  |  |  | **210** | **81** | **546** | **1,193** | **303** |  |
| S. coeruleoalba |  |  |  |  |  |  | **308** | **81** | **545** | **2,041** | **299** |  |
| L. albirostris |  |  |  |  |  |  | **242** | **81** | **546** | **1,431** | **296** |  |
| **O. orca** | **217** | **81** | **546** | **193** | **299** |  | **206** | **81** | **546** | **624** | **299** |  |
| **G. melas** | **150** | **80** | **na** | **75** | **285** |  | **249** | **80** | **536^1^** | **75** | **303** |  |

# Table S9. *IGF1* sites potentially associated with body size

Sites within the *IGF1* gene and flanking genomic regions that are significantly associated with body size in cetaceans, based on alignment of *Bos taurus* and 19 annotated cetacean IGF1 gene regions (see species in supplemental Figures S9, S10). “Basal toothed whales” refers to Orcinus orca (killer whale) and odontocete families Physeteridae, Kogiidae, and Ziphiidae.

| **Site type** | **Genomic coordinate in blue whale** | **Genomic feature in blue whale** | **Terrestrial artiodactyls** | **Small toothed whales** | **Killer whale** | **Baleen + basal toothed whales** | **Confirmed in the new alignment?** | **Exceptions** |
| --- | --- | --- | --- | --- | --- | --- | --- | --- |
| 1 | 85,108,387 | intron 3-4 | C | C | C | T | ? | *M. densirostris* has a gap |
| 1 | 85,118,402 | intron 2 | G | G | G | A | No | *P. phocoena* has A |
| 1 | 85,140,276 | intron 2 | C | C | C | T | No | New alignment inconsistent with old |
| 1 | 85,140,848 | intron 2 | A | A | A | G | No | *E. glacialis* has C |
| 1 | 85,150,447 | intron 2 | G | G | G | C | No | *E. glacialis* and *B. ricei* have G |
| 1 | 85,169,891 | intergenic, 5' of IGF1 | A | A | A | C | No | *E. glacialis* has A |
| 1 | 85,170,164 | intergenic, 5' of IGF1 | C | C | C | T | Yes |  |
| 2 | 85,112,779 | intron 3 | G | A | G | G | Yes |  |
| 2 | 85,126,807 | intron 2 | T | C | T | T | Yes |  |
| 2 | 85,132,164 | intron 2 | T | C | T | T | Yes |  |
| 2 | 85,160,446 | intron 2 | A | G | A | A | No | *E. glacialis* has T |
| 2 | 85,160,822 | intron 2 | T | C | T | T | Yes |  |

# Table S10. *IGF1* site SNV positions and nucleotides

Variants at each IGF1 SNV position from Bukhman et al. (2024) for each species in the sequence alignment. Species names are colored to match the size categories in Supplemental Figure S10 (red = small; blue = large). The positions in the sequence alignment file (IGF1_genomic_seqs.aln.gz; see https://osf.io/gq4ev/) are provided, along with the corresponding position in the blue whale genome sequence. Details of the alignment and site identification methods are provided in Bukhman et al. (2024).

|  |  | type 1 | type 1 | type 1 | type 1 | type 1 | type 2 | type 2 | type 2 | type 2 | type 2 | type 2 |
| --- | --- | --- | --- | --- | --- | --- | --- | --- | --- | --- | --- | --- |
| IGF1_genomic_seqs.aln column |  | 15354 | 15679 | 39141 | 51079 | 105222 | 25762 | 26152 | 69595 | 77606 | 90876 | 98108 |
| blue whale genome position |  | 85170164 | 85169891 | 85150447 | 85140848 | 85108387 | 85160822 | 85160446 | 85132164 | 85126807 | 85118402 | 85112779 |
| species | Mass |  |  |  |  |  |  |  |  |  |  |  |
| *Balaenoptera_musculus* | 136000 | T | C | C | G | T | T | A | T | T | G | G |
| *Balaenoptera_ricei* | 27216 | T | C | G | G | T | T | A | T | T | G | G |
| *Eschrichtius_robustus* | 23922 | T | C | C | G | T | T | A | T | T | G | G |
| *Eubalaena_glacialis* | 23000 | T | A | G | C | T | T | T | T | T | G | G |
| *Balaenoptera_acutorostrata* | 7500 | T | C | C | G | T | T | A | T | T | G | G |
| *Monodon_monoceros* | 1075 | C | A | G | A | C | C | G | C | C | A | A |
| *Pseudorca_crassidens* | 1054 | C | A | G | A | C | C | G | C | C | A | A |
| *Globicephala_melas* | 930 | C | A | G | A | C | C | G | C | C | A | A |
| *Delphinapterus_leucas* | 594 | C | A | G | A | C | C | G | C | C | A | A |
| *Lipotes_vexillifer* | 231 | C | A | G | A | C | C | G | C | C | A | A |
| *Lagenorhynchus_albirostris* | 180 | C | A | G | A | C | C | G | C | C | A | A |
| *Tursiops_truncatus* | 179 | C | A | G | A | C | C | G | C | C | A | A |
| *Neophocaena_asiaeorientalis_as* | 100 | C | A | G | A | C | C | G | C | C | A | A |
| *Delphinus_delphis* | 76 | C | A | G | A | C | C | G | C | C | A | A |
| *Phocoena_phocoena* | 53 | C | A | G | A | C | C | G | C | C | A | A |
| *Phocoena_sinus* | 43 | C | A | G | A | C | C | G | C | C | A | A |
| *Physeter_macrocephalus* | 32797 | T | C | C | G | T | T | A | T | T | G | G |
| *Orcinus_orca* | 3018 | C | A | G | A | C | T | A | T | T | A | G |
| *Mesoplodon_densirostris* | 1069 | T | A | G | A | G | T | A | T | T | A | G |
| *Kogia_breviceps* | 345 | T | C | G | A | T | T | A | T | T | G | G |
| *Bos_taurus* |  | C | A | G | A | C | T | A | T | T | G | G |
| *Canis_lupus_familiaris* |  | C | A | G | A | C | T | A | T | T | G | G |
| *Homo_sapiens* |  | T | A | C | A | T | T | A | - | T | A | A |

# Table S11. VCF file information

List of variant call format (VCF) files for based on whole genome resequencing data (Table S3) mapped to the reference for each species. Variants sites were called and filtered using DeepVariant v1.6.0 as described in the methods.

| Species | Latin name | Reference assembly | BioSample ID | VCF file | No. of biallelic SNPs (M) |
| --- | --- | --- | --- | --- | --- |
| Common minke whale | *Balaenoptera acutorostrata* | GCA_949987535.1 | [SAMEA111380540](https://www.ncbi.nlm.nih.gov/biosample/SAMEA111380540/) | ERR11040176_GCA_949987535.1_mBalAcu1.1.deepvariant.vcf.gz | 1.057 |
| Blue whale | *Balaenoptera musculus* | GCA_009873245.3 | [SAMN07201754](https://www.ncbi.nlm.nih.gov/biosample/SAMN07201754) | SRR5665644_GCF_009873245.2_mBalMus1.pri.v3_dedup.deepvariant.vcf.gz | 8.403 |
| Rice's whale | *Balaenoptera ricei* | GCA_028023285.1 | [SAMN13072001](https://www.ncbi.nlm.nih.gov/biosample/SAMN13072001) | SRR10331559_GCA_028023285.1_mBalRic1.hap2_dedup.deepvariant.vcf.gz | 0.3691 |
| Common dolphin | *Delphinus delphis* | GCA_949987515.1 | [SAMEA111380534](https://www.ncbi.nlm.nih.gov/biosample/SAMEA111380534/) | ERR11040185_GCA_949987515.1_mDelDel1.1_dedup.deepvariant.vcf.gz | 3.245 |
| Gray whale | *Eschrichtius robustus* | GCA_028021215.1 | [SAMN15801458](https://www.ncbi.nlm.nih.gov/biosample/SAMN15801458) | SRR12437599_GCA_028021215.1_mEscRob2.pri_dedup.deepvariant.vcf.gz | 1.828 |
| North Atlantic right whale | *Eubalaena glacialis* | GCA_028564815.1 | [SAMN14122067](https://www.ncbi.nlm.nih.gov/biosample/SAMN14122067) | SRR11097130_GCA_028564815.1_mEubGla1_dedup.deepvariant.vcf.gz | 0.758 |
| Long-finned pilot whale | *Globicephala melas* | GCA_963455315.1 | [SAMEA111380538](https://www.ncbi.nlm.nih.gov/biosample/SAMEA111380538/) | ERR11837528_GCA_963455315.1_mGloMel1.1_dedup.deepvariant.vcf.gz | 1.579 |
| Northern bottlenose whale | *Hyperoodon ampullatus* | GCA_949752795.1 | [SAMEA10839125](https://www.ncbi.nlm.nih.gov/biosample/SAMEA10839125/) | ERR10908646_GCA_949752795.1_mHypAmp2.1_dedup.deepvariant.vcf.gz | 1.195 |
| Amazon River dolphin | *Inia geoffrensis* | GCA_036417435.1 | SAMN07678125 | SRR11430609_GCA_036417435.1_mIniGeo1_dedup.deepvariant.vcf.gz | 2.722 |
| Pygmy sperm whale | *Kogia breviceps* | GCA_026419985.1 | [SAMN07678126](https://www.ncbi.nlm.nih.gov/biosample/SAMN07678126) | SRR11430611_GCA_026419985.1_mKogBre1_dedup.deepvariant.vcf.gz | 20.29 |
| White-beaked dolphin | *Lagenorhynchus albirostris* | GCA_949774975.1 | SAMEA111380547 | ERR11042964_GCA_949774975.1_mLagAlb1.1_dedup.deepvariant.vcf.gz | 0.506 |
| Blainville's beaked whale | *Mesoplodon densirostris* | GCA_025265405.1 | [SAMN16895766](https://www.ncbi.nlm.nih.gov/biosample/SAMN16895766) | SRR13167975_GCF_025265405.1_mMesDen1_dedup.deepvariant.vcf.gz | 5.521 |
| East Asian finless porpoise | *Neophocaena asiaeorientalis sunameri* | GCA_026225855.1 | SAMN30090519 | SRR21047154_GCA_026225855.1_NeoAsi_dedup.deepvariant.vcf.gz | 2.786 |
| Killer whale | *Orcinus orca* | GCA_937001465.1 | SAMN01180276 | SRR574970_98_merged_GCA_937001465.1_mOrcOrc1.1_dedup.deepvariant.vcf.gz | 1.08 |
| Harbor porpoise | *Phocoena phocoena* | GCA_963924675.1 | SAMN07678060 | SRR11432011_GCA_963924675.1_mPhoPho1.1_dedup.deepvariant.vcf.gz | 6.924 |
| Vaquita | *Phocoena sinus* | GCA_008692025.1 | [SAMN20563478](https://www.ncbi.nlm.nih.gov/biosample/SAMN20563478) | SRR15435906_GCA_008692025.1_mPhoSin1.pri_dedup.deepvariant.vcf.gz | 0.3892 |
| Striped dolphin | *Stenella coeruleoalba* | GCA_951394435.1 | [SAMEA111380539](https://www.ncbi.nlm.nih.gov/biosample/SAMEA111380539/) | ERR11042965_GCA_951394435.1_mSteCoe1.1_dedup.deepvariant.vcf.gz | 7.006 |
| Common bottlenose dolphin | *Tursiops truncatus* | GCA_011762595.1 | SAMN12611942 | pat18819988_GCF_011762595.1_mTurTru_dedup.deepvariant.vcf.gz | 3.096 |

# Figure S1. Consensus nuclear locus phylogeny

# Figure S2. Ancestral linkage group karyotypes

The predicted karyotypes for all species with apparent fusion events, and extant chromosome paintings based on the ALGs. Species names are given in Table 1 (main text), plus ancestral outgroups *Bos Taurus* (TBtau) and *Hippopotamus amphibius* (HypAmp2).


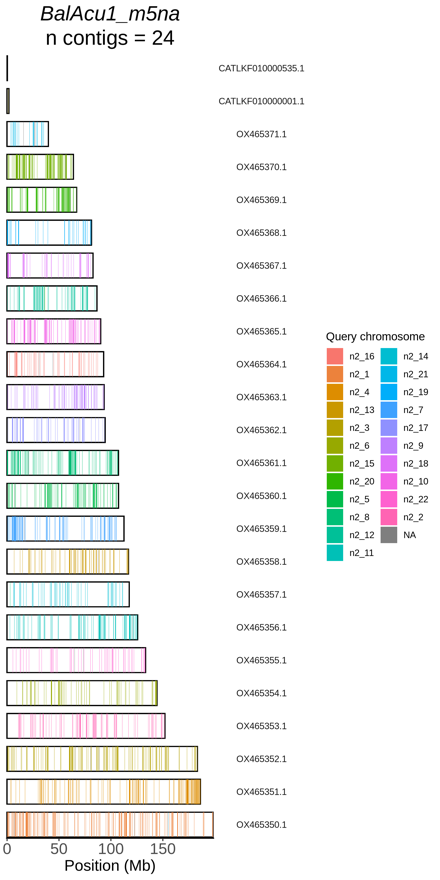

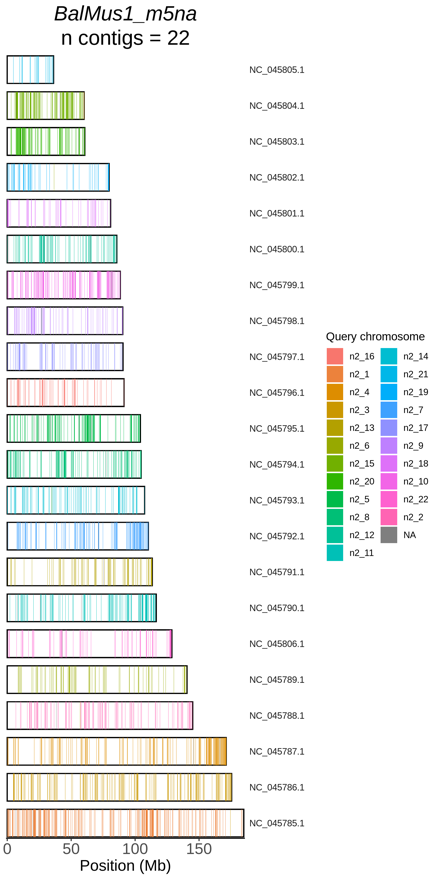

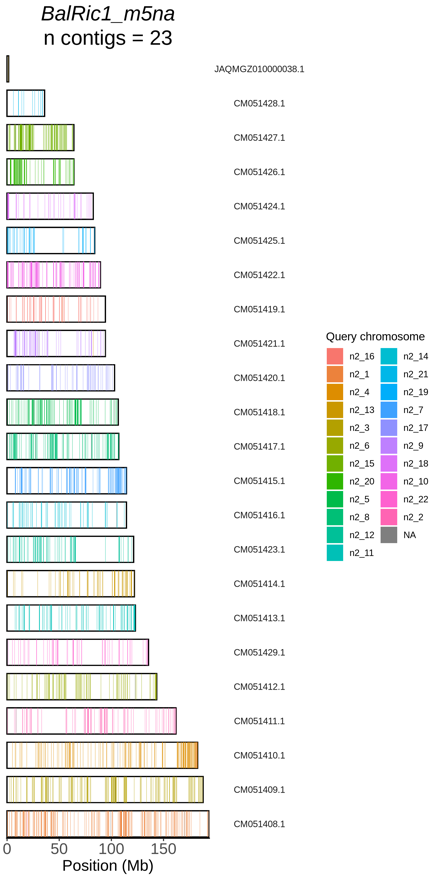

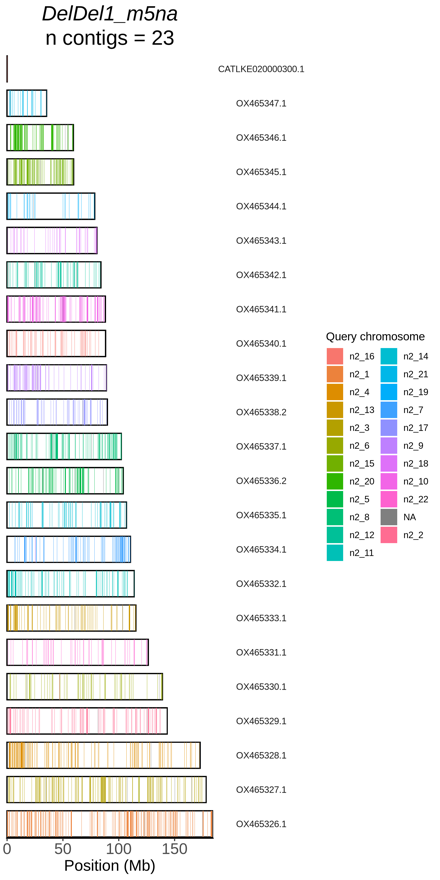


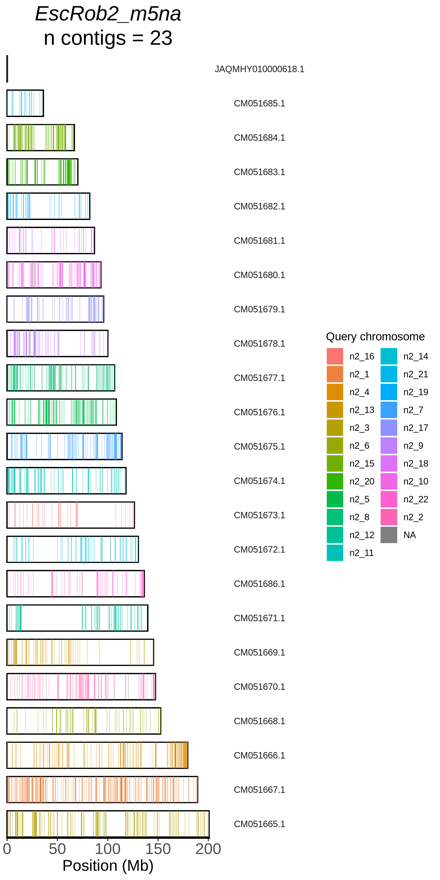

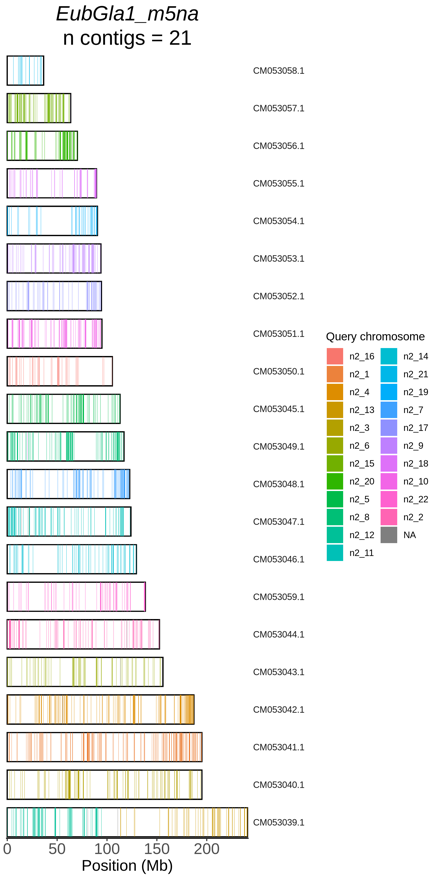

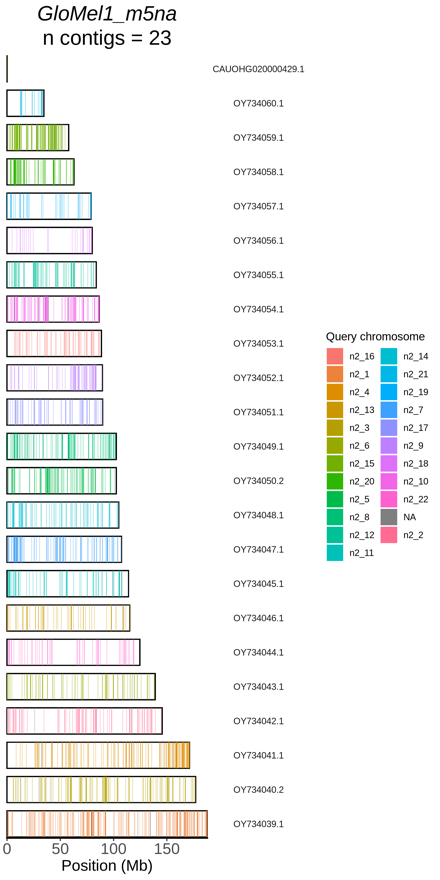

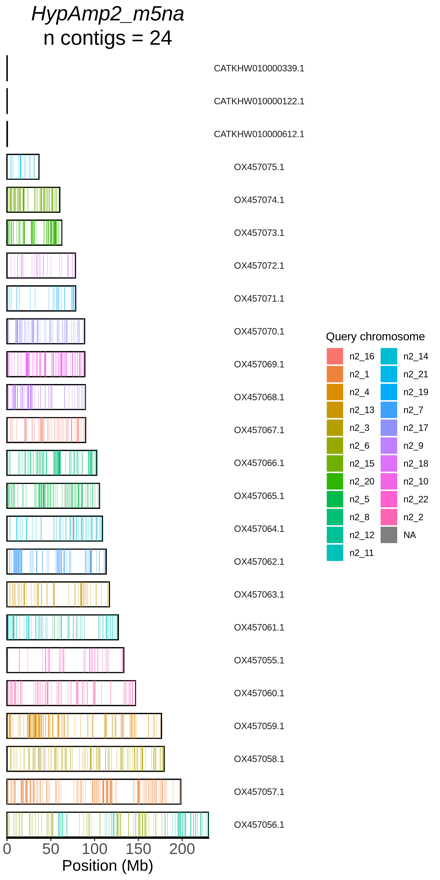

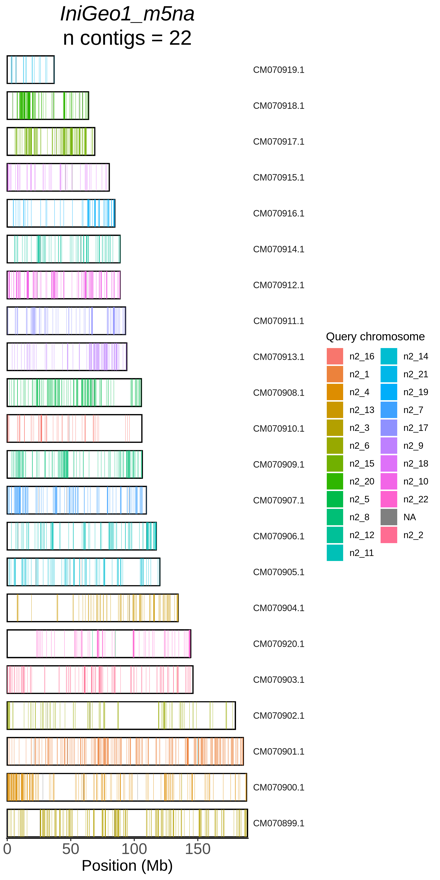

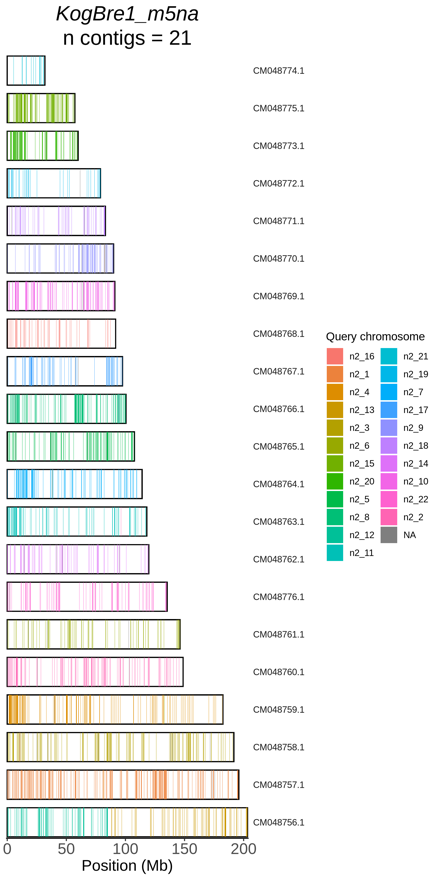

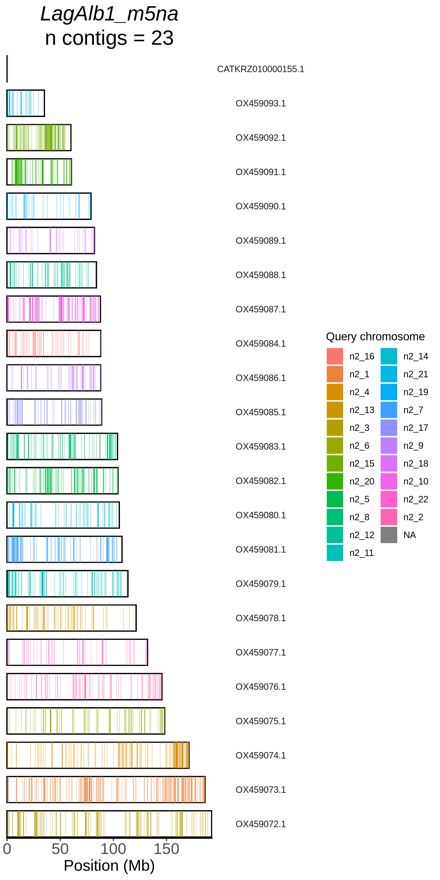

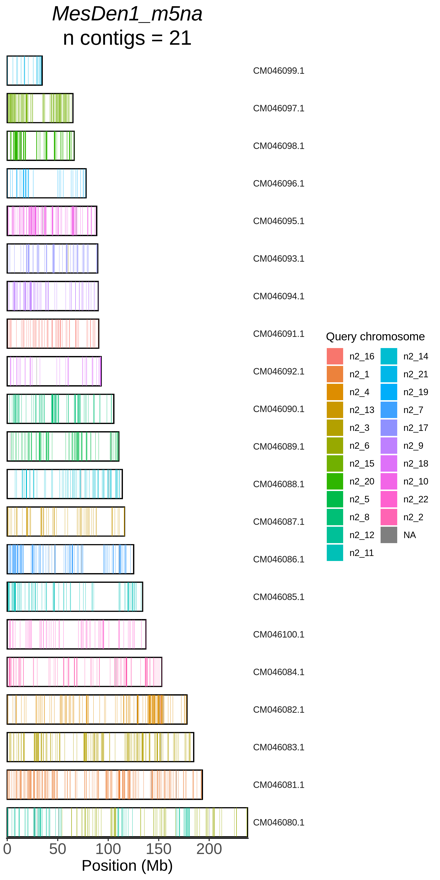

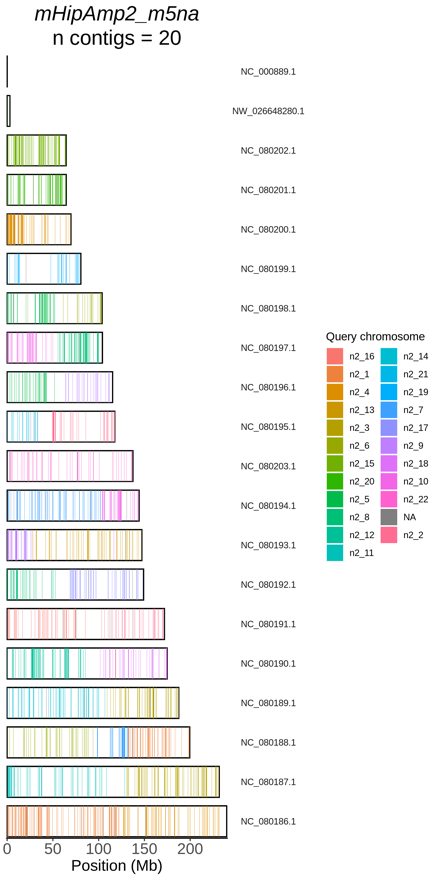

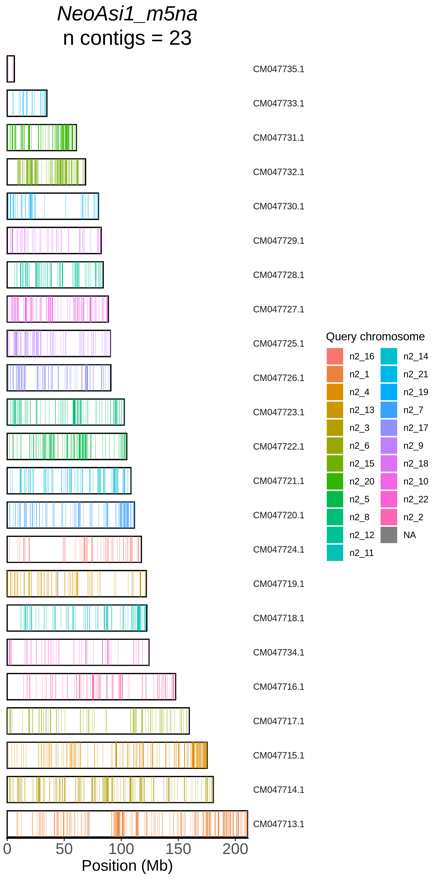

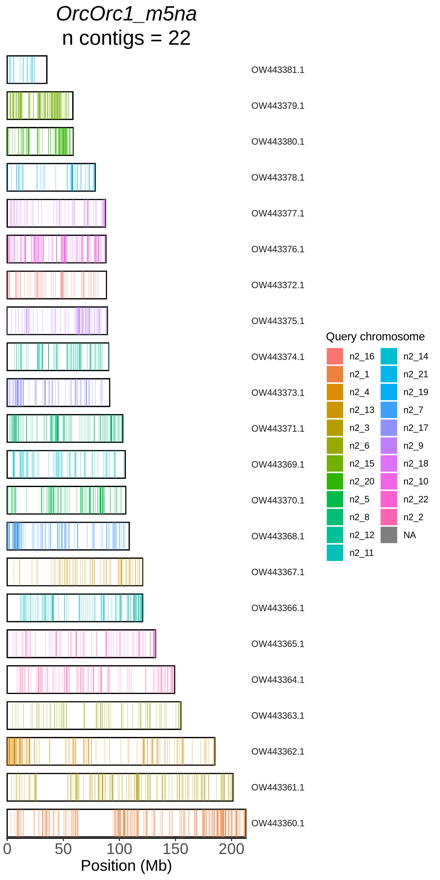

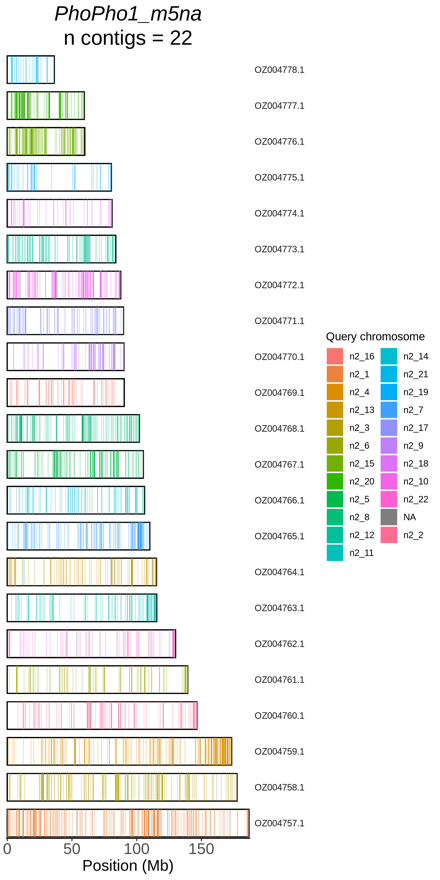

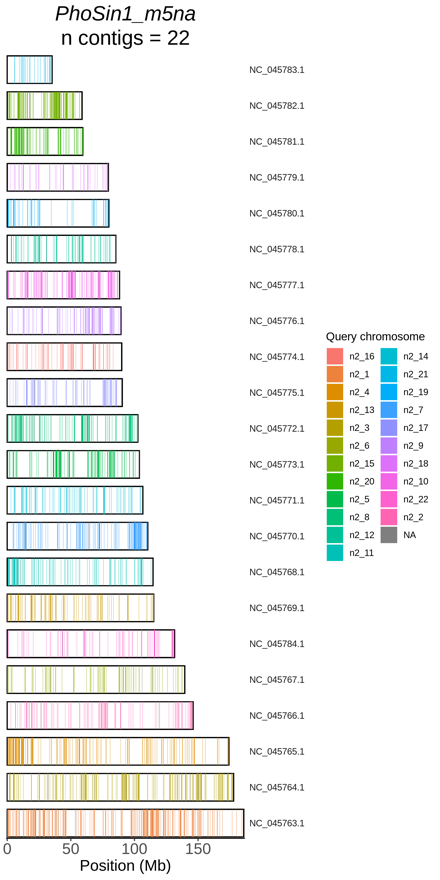

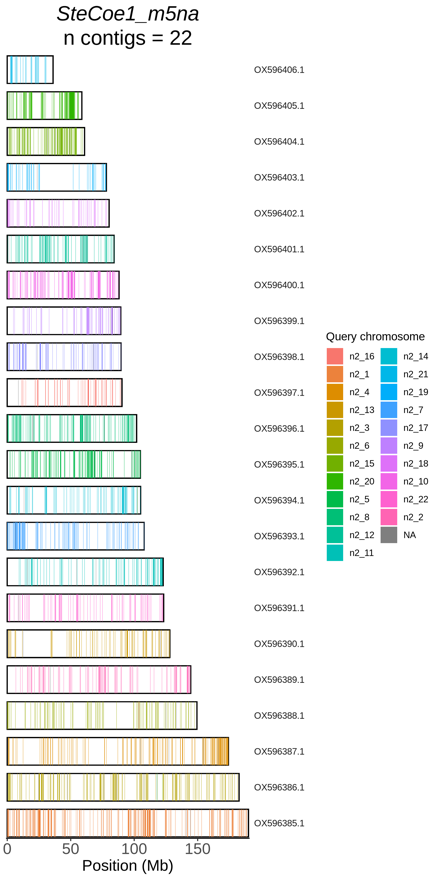

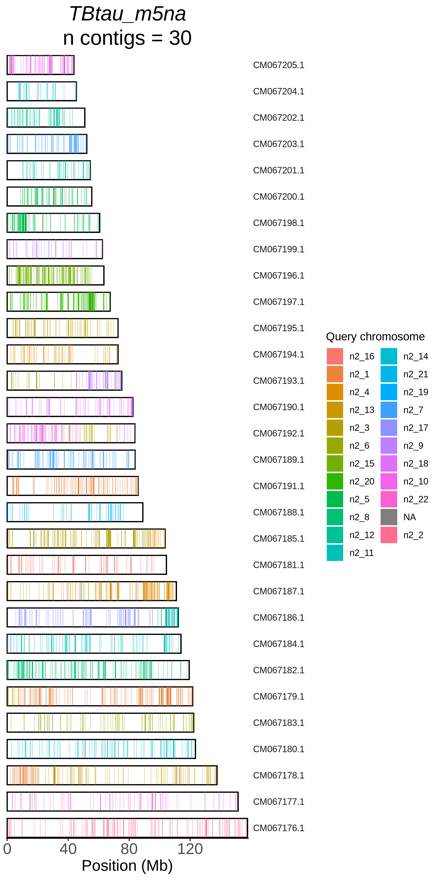

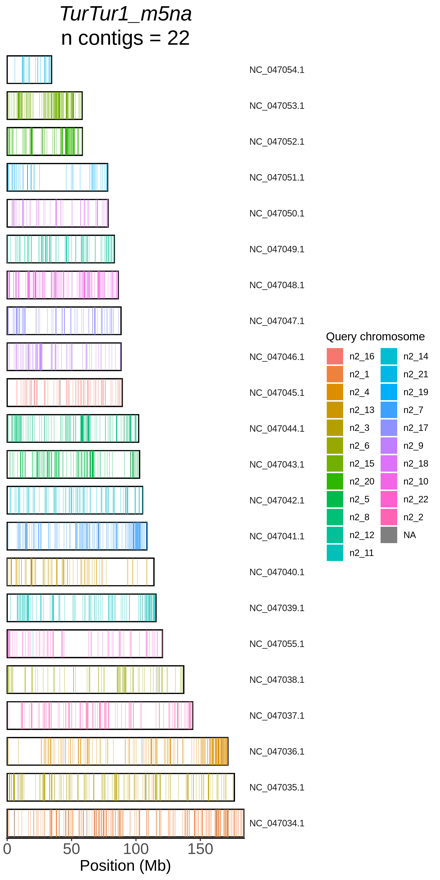


# Figure S3. Genome annotation summary

Count of Genes (including pseudogenes) and coding sequences (CDSs) for annotated reference genomes, from the NCBI RefSeq annotation release of each genome (as of August 14, 2024).

# Figure S4. Repeat landscape profile comparison

Repeat landscape profiles reference genomes except *O. orca* and *D. delphis* (see main text, Figure 4), based on repeat masking with RepeatMasker 4.6.1 using Dfam library 3.8.


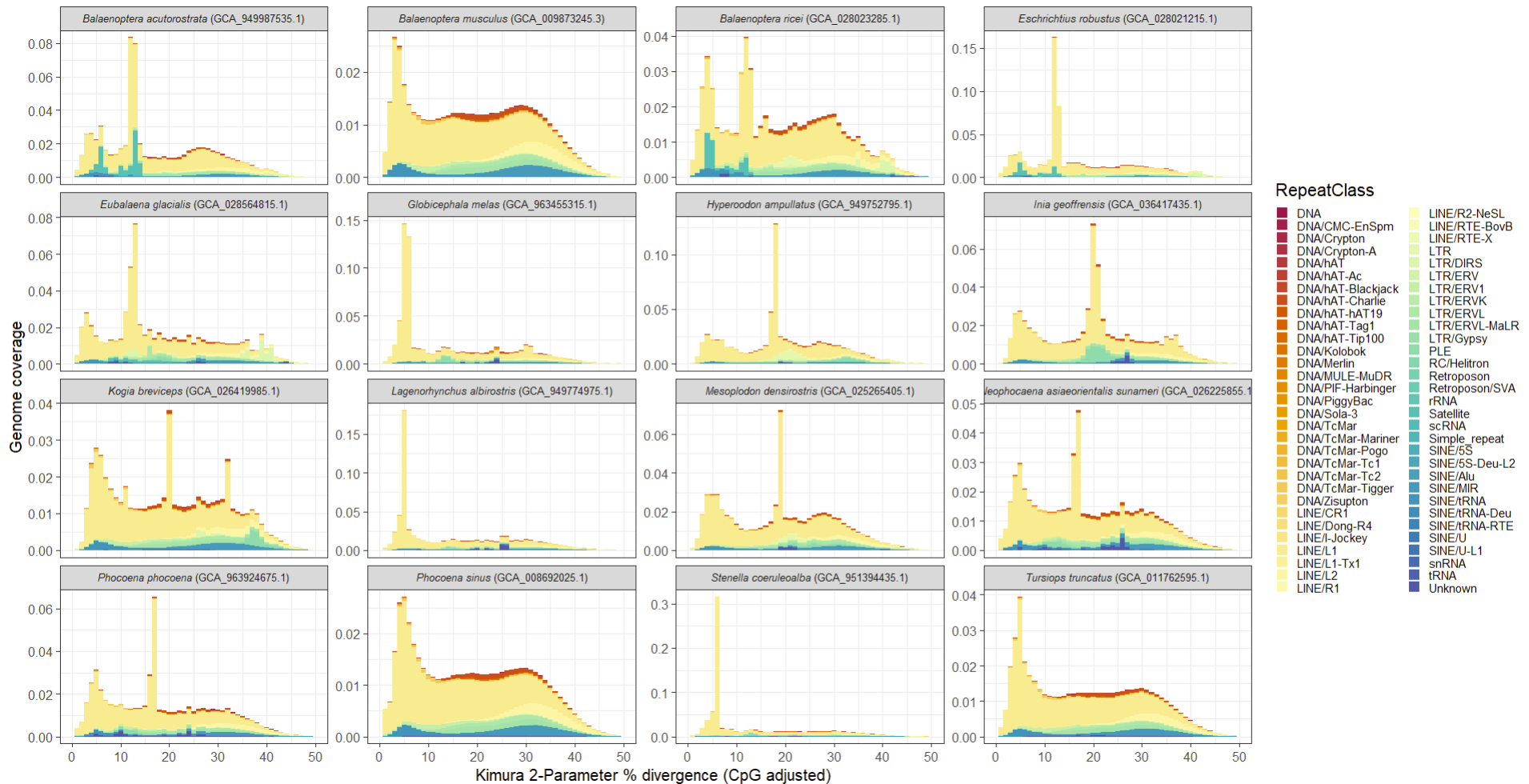


# Figure S5. Heterozygosity per 1MB window

Individual plots of heterozygosity (heterozygotes per 1000bp) in 1MB non-overlapping windows for all autosomes.

Common minke whale (*Balaenoptera acutorostrata*) Blue whale (*Balaenoptera musculus*)

Rice's whale (*Balaenoptera ricei*) Common (saddleback) dolphin (*Delphinus delphis*)

Gray whale (*Eschrichtius robustus*) North Atlantic right whale (*Eubalaena glacialis*)

Long-finned pilot whale (*Globicephala melas*) Northern bottlenose whale (*Hyperoodon ampullatus*)

Amazon River dolphin (*Inia geoffrensis*) Pygmy sperm whale (*Kogia breviceps*)

White-beaked dolphin (*Lagenorhynchus albirostris*) Blainville's beaked whale (*Mesoplodon densirostris*)

East Asian finless porpoise (*Neophocaena asiaeorientalis*) Killer whale (*Orcinus orca*)

Harbor porpoise (*Phocoena phocoena*) Vaquita (*Phocoena sinus*)

Striped dolphin (*Stenella coeruleoalba*) Common bottlenose dolphin (*Tursiops truncatus*)

# Figure S6. Genome coverage comparison

Coverage distribution along chromosome 1 (repeats not masked) for all reference genome species. Short read data for the minke whale, common dolphin, long-finned pilot whale, northern bottlenose whale, white-beaked common dolphin, and striped dolphin were Hi-C sequences, whereas short read data for all other species were whole genome shotgun sequences.

Minke whale (*Balaenoptera acutorostrata*)

Blue whale (*Balaenoptera musculus*)

Rice's whale (*Balaenoptera ricei*)

Common dolphin (*Delphinus delphis*)

Gray whale (*Eschrichtius robustus*)

N. Atlantic right whale (*Eubalaena glacialis*)

Long-finned pilot whale (*Globicephala melas*)

Northern bottlenose whale (*Hyperoodon ampullatus*)

Amazon River dolphin (*Inia geoffrensis*)

Pygmy sperm whale (*Kogia breviceps*)

White-beaked dolphin (*Lagenorhynchus albirostris*)

Blainville's beaked whale (*Mesoplodon densirostris*)

East Asian finless porpoise (*Neophocaena asiaeorientalis*)

Killer whale (*Orcinus orca*)

Harbor porpoise (*Phocoena phocoena*)

Vaquita (*Phocoena sinus*)

Striped dolphin (*Stenella coeruleoalba*)

Common bottlenose dolphin (*Tursiops truncatus*)

# Figure S7. Historical demography plots

Individual plots of historical demography based on PSMC. Sequence SRA data (Table S1) were mapped to the reference genome of each species, repeats removed, and PSMC was run with species-specific generation times (g; Table S4). Mutation rate was 4.90E-10 mutations/site/yr, scaled to generation time for each species. All other parameters were held constant (see methods). Note that the axis scales are not fixed.

Common minke whale (*Balaenoptera acutorostrata*) Blue whale (*Balaenoptera musculus*)

Rice's whale (*Balaenoptera ricei*) Common (saddleback) dolphin (*Delphinus delphis*)

Gray whale (*Eschrichtius robustus*) North Atlantic right whale (*Eubalaena glacialis*)

Long-finned pilot whale (*Globicephala melas*) Northern bottlenose whale (*Hyperoodon ampullatus*)

Amazon River dolphin (*Inia geoffrensis*) Pygmy sperm whale (*Kogia breviceps*)

White-beaked dolphin (*Lagenorhynchus albirostris*) Blainville's beaked whale (*Mesoplodon densirostris*)

East Asian finless porpoise (*Neophocaena asiaeorientalis*) Killer whale (*Orcinus orca*)

Harbor porpoise (*Phocoena phocoena*) Vaquita (*Phocoena sinus*)

Striped dolphin (*Stenella coeruleoalba*) Common bottlenose dolphin (*Tursiops truncatus*)

# Figure S8: MHC class I and class IIa assembly improvements

Class I regions from draft (short-read; DNAzoo) and reference (long-read) assemblies derived from the same individual were compared in a Mauve alignment. Top sequence represents reference, bottom sequence the draft assembly. Length of sequence is shown in kb. Blocks of similar sequences are shown in different colors; when the color block is underneath the black line, the sequence is reversed at this position, when no block is present, that part of the sequence is missing in the other. The following genes are shown below each sequence: representative framework genes (green) TRIM26 (tripartite motif containing 26) and ABCF1 (ATP-binding cassette subfamily F member1), TCF19 (transcription factor19) and DXX39B (DExD-box helicase 39B). HLA-B like genes in white, blue genes BoLA-like class I genes. Positions of genes indicative only.


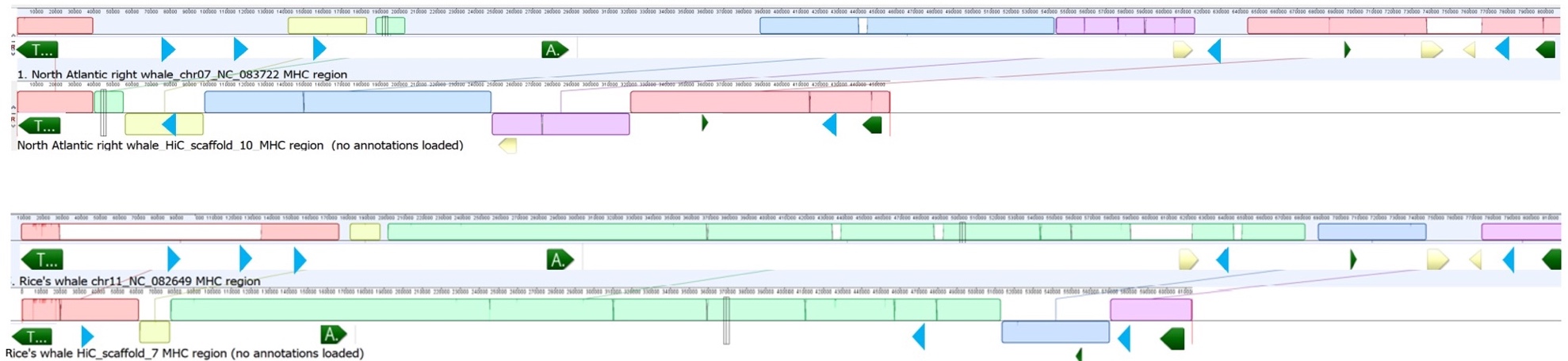


# Figure S9. IGF1 gene type 1 SNV correlation with body size

A typical IGF1 type 1 site (blue whale genome coordinate chr.10: 85,169,891). Compare to Bukhman et al. 2024 Figure 5b - d. A. Cetacean body mass vs. SNV allele. B. Distribution of alternative alleles on the phylogenetic tree (TimeTree). Only annotated genomes were included in the analysis. Analysis methods were previously described (Bukhman et al., 2024), and cetacean body mass values were retrieved from Groot et al. (2023) and other sources as specified in the R Markdown notebook from Bukhman et al. 2024.

A B

# Figure S10. IGF1 gene type 2 SNV correlation with body size

A typical IGF1 type 2 site (blue whale genome coordinate chr.10:85,160,822). Compare to Bukhman et al. 2024 Figure 5b - d. A. Cetacean body mass vs. SNV allele. B. Distribution of alternative alleles on the phylogenetic tree (TimeTree). Only annotated genomes were included in the analysis. Analysis methods were previously described (Bukhman et al., 2024), and cetacean body mass values were retrieved from Groot et al. (2023) and other sources as specified in the R Markdown notebook from Bukhman et al. 2024.

A B

# Figure S11: The MHC region from GABBR1 gene to ELOVL5

The MHC region from GABBR1 gene to ELOVL5 and GC content in %, comprising class I, class III, and class IIa, for all 18 cetacean species with a high-quality reference genome. The length of the genomic sequence is indicated, and representative genes for framework genes are marked in green (Representative framework genes were selected from human MHC (HLA) as presented in Shiina et al (2017). This graphic does not contain any MHC genes. The GC % content is indicated in a graph below each sequence (GC blue, AT green) for a sliding 500bp window.

E. glacialis


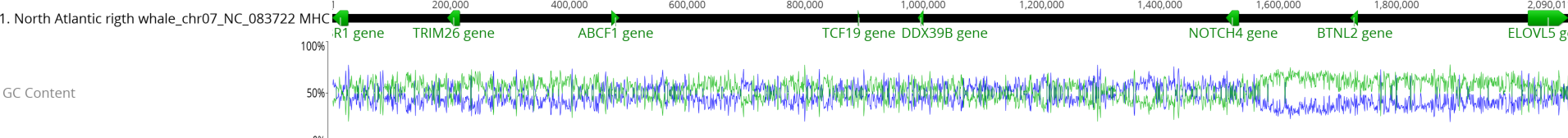


B. acutorostrata


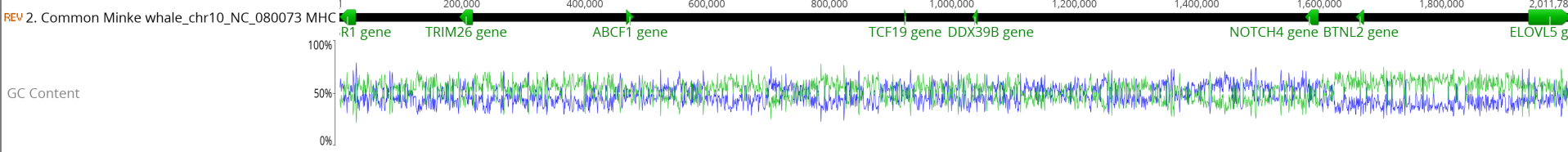


E. robustus


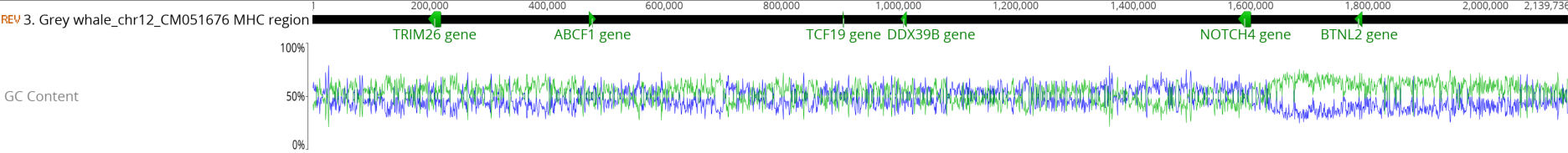


B. musculus


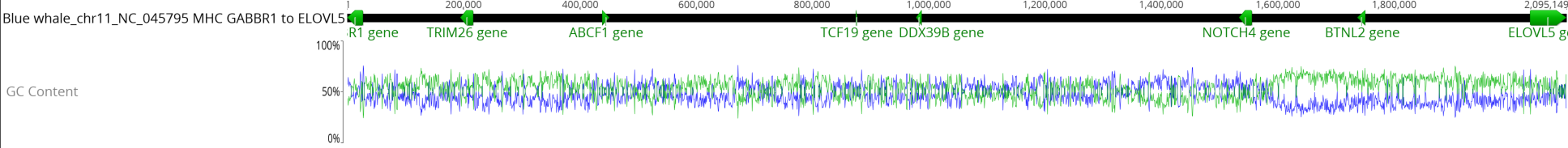


B. ricei


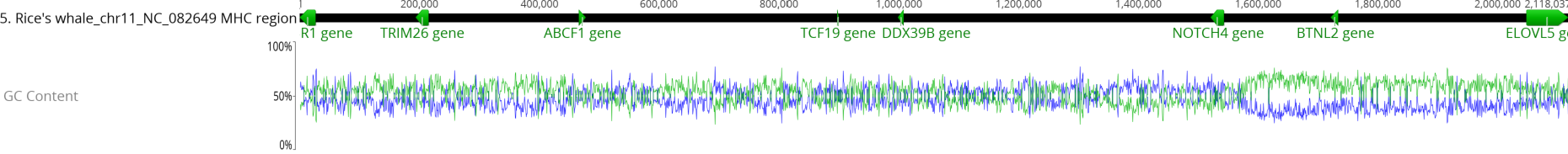


K. breviceps


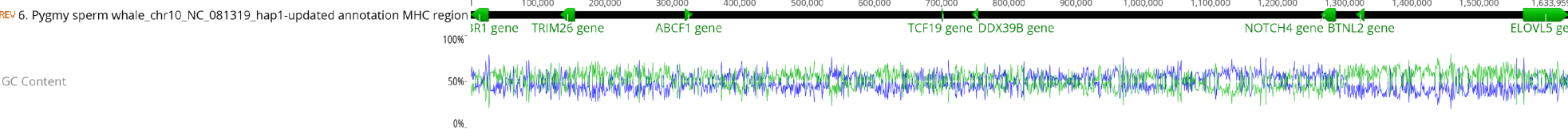


H. ampullatus


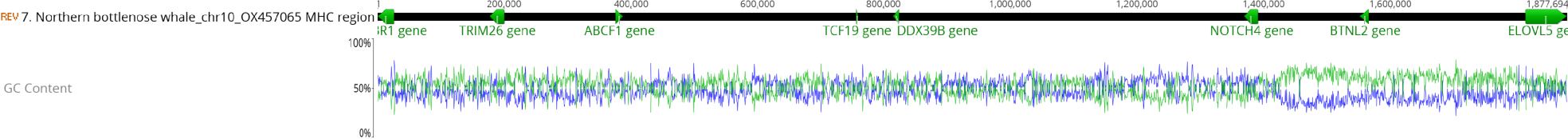


M. densirostris


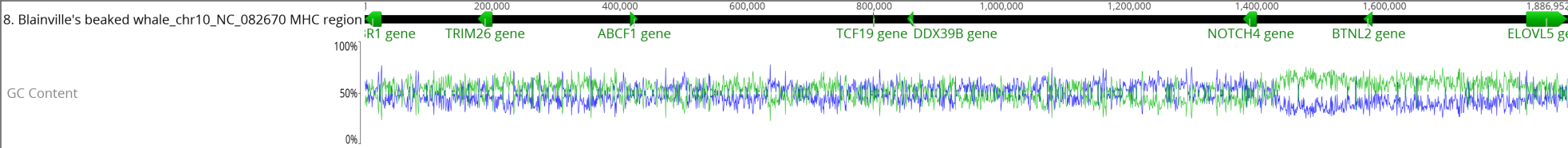


I. geoffrensis


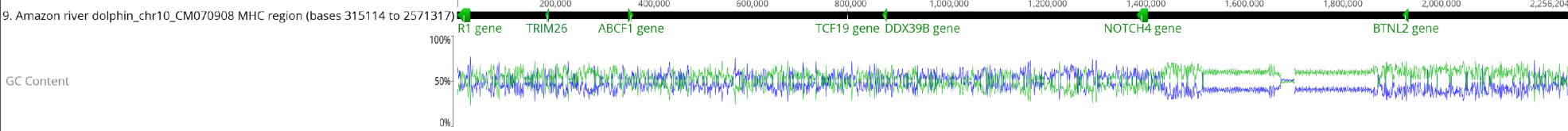


N. asiaorientalis


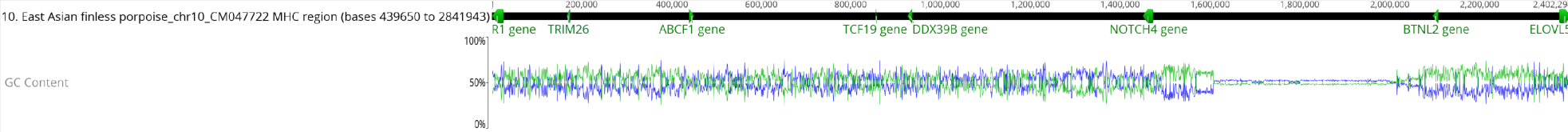


P. sinus


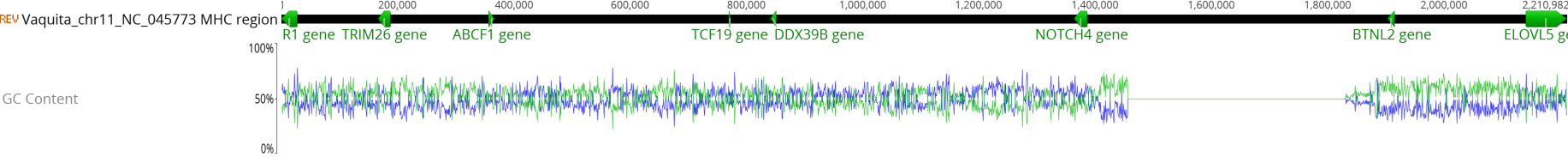


P. phocaena


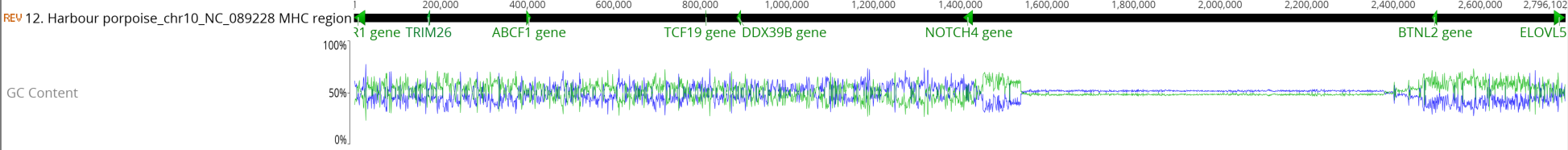


T. truncatus


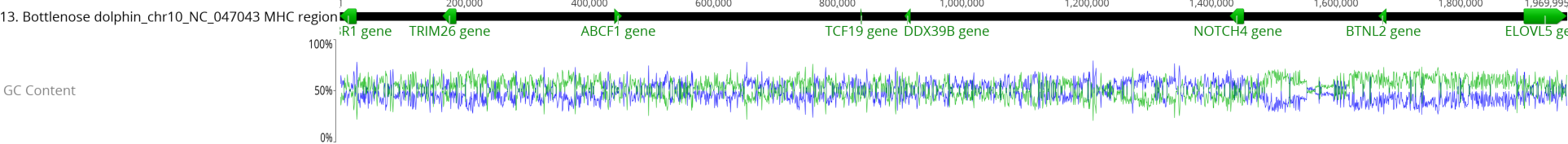


D. delphis


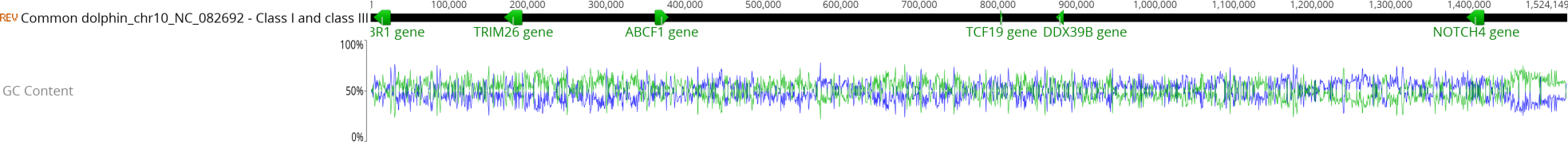


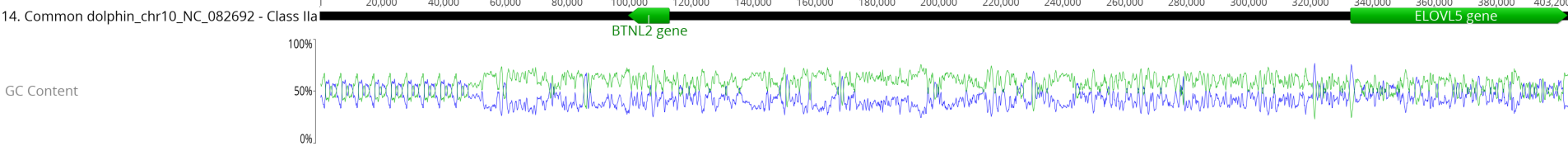


S. coeruleoalba


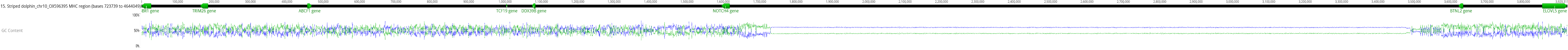


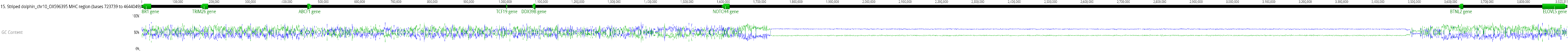


L. albirostris


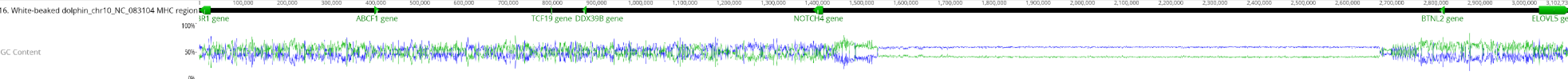


O. orca


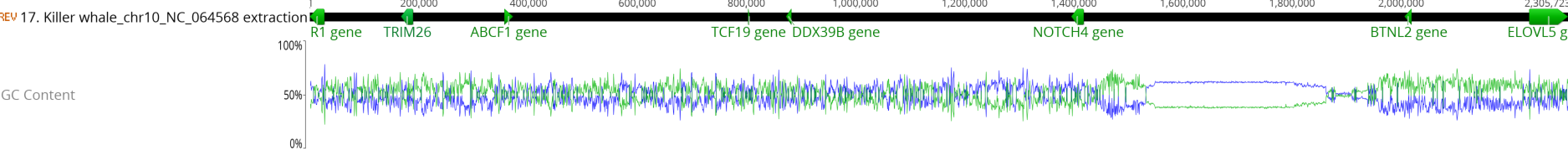


G. melas


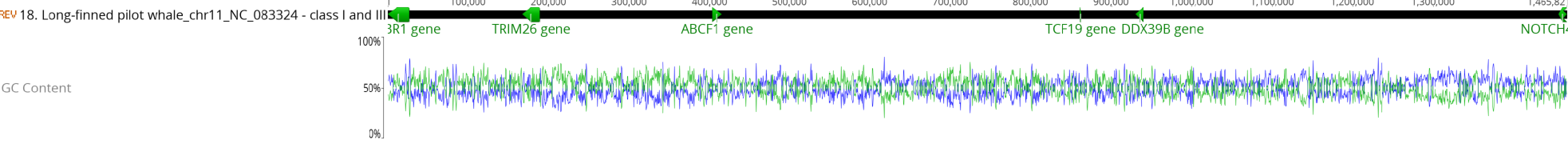

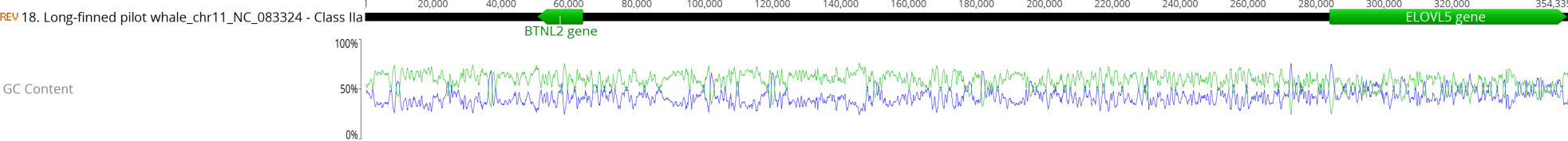


# References

Arnold, K., Gosling, J., and Holmes, D. (2005). *The Java Programming Language, Forth Edition.* Addison Wesley Professional.

Bonfield, J.K., Marshall, J., Danecek, P., Li, H., Ohan, V., Whitwham, A., et al. (2021). HTSlib: C library for reading/writing high-throughput sequencing data. *Gigascience* 10. doi: 10.1093/gigascience/giab007

Bukhman, Y.V., Morin, P.A., Meyer, S., Chu, L.-F., Jacobsen, J.K., Antosiewicz-Bourget, J., et al. (2024). A high-quality blue whale genome, segmental duplications, and historical demography. *Molecular Biology and Evolution* 41**,** msae036. doi: 10.1093/molbev/msae036

Cheng, H., Concepcion, G.T., Feng, X., Zhang, H., and Li, H. (2021). Haplotype-resolved de novo assembly using phased assembly graphs with hifiasm. *Nat Methods* 18**,** 170-175. doi: 10.1038/s41592-020-01056-5

Cheng, H., Jarvis, E.D., Fedrigo, O., Koepfli, K.P., Urban, L., Gemmell, N.J., et al. (2022). Haplotype-resolved assembly of diploid genomes without parental data. *Nat Biotechnol* 40**,** 1332-1335. doi: 10.1038/s41587-022-01261-x

Danecek, P., Bonfield, J.K., Liddle, J., Marshall, J., Ohan, V., Pollard, M.O., et al. (2021). Twelve years of SAMtools and BCFtools. *Gigascience* 10**,** giab008. doi: 10.1093/gigascience/giab008

Dierckxsens, N., Mardulyn, P., and Smits, G. (2017). NOVOPlasty: assembly of organelle genomes from whole genome data. *Nucleic Acids Research* 45. doi: ARTN e18

10.1093/nar/gkw955

Groot, N.E., Constantine, R., Garland, E.C., and Carroll, E.L. (2023). Phylogenetically controlled life history trait meta-analysis in cetaceans reveals unexpected negative brain size and longevity correlation. *Evolution* 77**,** 534-549. doi: 10.1093/evolut/qpac050

Guan, D., McCarthy, S.A., Wood, J., Howe, K., Wang, Y., and Durbin, R. (2020). Identifying and removing haplotypic duplication in primary genome assemblies. *Bioinform* 36**,** 2896-2898. doi: 10.1093/bioinformatics/btaa025

Li, H. (2013). Aligning sequence reads, clone sequences and assembly contigs with BWA-MEM. *arXiv: Genomics*. doi:

Li, H. (2018). Minimap2: pairwise alignment for nucleotide sequences. *Bioinform* 34**,** 3094-3100. doi: 10.1093/bioinformatics/bty191

Li, H. (2021). New strategies to improve minimap2 alignment accuracy. *Bioinform* 37**,** 4572-4574. doi: 10.1093/bioinformatics/btab705

Poplin, R., Chang, P.C., Alexander, D., Schwartz, S., Colthurst, T., Ku, A., et al. (2018). A universal SNP and small-indel variant caller using deep neural networks. *Nat Biotechnol* 36**,** 983-987. doi: 10.1038/nbt.4235

Shiina, T., Blancher, A., Inoko, H., and Kulski, J.K. (2017). Comparative genomics of the human, macaque and mouse major histocompatibility complex. *Immunology* 150**,** 127-138. doi: 10.1111/imm.12624

Taylor, B.L., Chivers, S.J., Larese, J., and Perrin, W.F. (2007). "Generation length and percent mature estimates for IUCN assessments of cetaceans". Southwest Fisheries Science Center, 8604 La Jolla Shores Blvd., La Jolla, CA 92038, USA).

Zhou, C., McCarthy, S.A., and Durbin, R. (2023). YaHS: yet another Hi-C scaffolding tool. *Bioinform* 39. doi: 10.1093/bioinformatics/btac808
